# Explainable AI for Network Pharmacology: A Critical Framework for Interpreting Multi-Target Drug Discovery on Biological Graphs

**DOI:** 10.3390/ijms27219479

**Published:** 2026-10-23

**Authors:** Khaled S. Allemailem, Ahmad Almatroudi, Abdullah M. Alnajim

**Affiliations:** 1 Department of Medical Laboratories, College of Applied Medical Sciences, Qassim University, Buraydah 51452, Saudi Arabia; aamtrody@qu.edu.sa; 2 Department of Information Technology, College of Computer, Qassim University, Buraydah 51452, Saudi Arabia; najim@qu.edu.sa

**Keywords:** explainable AI, network pharmacology, graph neural networks, knowledge graphs, multi-target drug discovery, polypharmacology, interpretability, SHAP, GNNExplainer, causal explainability

## Abstract

Network pharmacology examines drug action across interacting targets, pathways, and disease modules. Increasingly, however, the graph neural networks and knowledge-graph models used for this purpose are difficult to inspect. This critical narrative review considers feature attribution, attention, counterfactual and rule-based explanation, explainable graph neural networks, Bayesian and causal methods, and path-based knowledge-graph reasoning. We assess these approaches against three demands imposed by biological graphs: preservation of heterogeneous node and relation semantics, support for multi-hop reasoning, and concordance with evidence excluded from model training. Among the methods reviewed, none has yet been shown to satisfy all three demands consistently. A model-faithful explanation may still emphasize node degree, network hubs, or heavily studied entities instead of a disease-specific mechanism. To address this problem, we propose five evaluation domains: faithfulness, stability, external biological concordance, bias sensitivity, and translational utility, together with a six-item reporting standard. Both are conceptual proposals; they remain unweighted, uncalibrated, and unevaluated empirically. An exploratory, literature-based application to TxGNN, DDI-GCN, and MINERVA shows that transparency-related reporting is useful but incomplete. This exercise demonstrates how the criteria might be used; it is not an empirical evaluation or calibration of the framework. We did not retrain or rerun any model or conduct new quantitative benchmarking. The field still lacks a unified biomedical benchmark that evaluates prediction and explanation together under heterogeneous, cold-start, and provenance-aware graph settings. Progress will depend on task-matched evaluation, explicit treatment of uncertainty, and experiments designed to distinguish what a model uses from what biology actually does.

## 1. Introduction

### 1.1. The Multi-Target Imperative in Modern Drug Discovery

Complex disease rarely reduces to the failure of one protein. Cancer signaling, neurodegeneration, metabolic syndrome, and inflammatory disease involve redundant pathways, cross-talk, and feedback loops; blocking one node may therefore redirect signaling through another route [1]. Such redundancy can promote off-target effects, compensatory resistance, and the failure of otherwise plausible single-target interventions. The importance of each mechanism depends on the disease, target, and stage of development, so it should not be summarized by a single universal failure rate.

Polypharmacology, the deliberate modulation of several targets by one agent or a combination, has shifted from an unwanted complication to a design strategy. Recent work places this shift within network pharmacology, supported by AI methods and large biomedical knowledge graphs [2]. Clinically successful multi-target kinase inhibitors and epigenetic modulators illustrate the potential of network-aware intervention, while also showing how difficult it remains to design such activity prospectively.

### 1.2. Why Network Pharmacology Needs AI and Why That AI Needs to Be Explainable

Network pharmacology represents genes, proteins, metabolites, and phenotypes as connected systems, with hubs, bottlenecks, and modules serving as potential points of therapeutic leverage. Modern biomedical graphs now exceed the scale at which manual inspection or conventional graph statistics are sufficient. Machine-learning and graph-neural-network models are consequently used for drug–target prediction, protein-interaction inference, disease–gene association, drug repositioning, and multi-target prioritization.

These models can be highly predictive, but predictive accuracy does not make their reasoning transparent. A drug–target prediction is of limited mechanistic value if the model cannot identify the molecular features, graph neighborhood, or multi-hop path that influenced it. This distinction matters in network pharmacology because the scientific objective is not only to rank candidates, but also to formulate explanations that medicinal chemists, systems pharmacologists, and experimental researchers can test.

Explainable AI (XAI) offers several ways to examine these models. SHAP, LIME, saliency mapping, counterfactual reasoning and GNNExplainer each expose something about how a model behaves, and what they expose can be weighed against what medicinal chemists already know, which is often enough to suggest a mechanistic hypothesis worth following up. How far any of this has matured depends heavily on the data modality [3]. Work on network-structured biomedical data lags behind work on molecular graphs and conventional QSAR tasks.

### 1.3. Positioning and Contribution

Three recent reviews come close enough to the present topic that a direct comparison is worth making.

Li et al. [2], in npj Precision Oncology, trace the field from network biology through machine learning, graph neural networks and knowledge graphs on to generative and reinforcement-learning approaches, experimental evaluation with CETSA, NanoBRET and Perturb-seq, and finally clinical translation. Explainability enters that account as one contributor to trust and adoption among several. Theirs is probably the most comprehensive overview of AI-augmented network pharmacology published so far, but it is not primarily a methodological treatment of explainability: SHAP and attention are discussed briefly, and graph-specific explainers such as GNNExplainer, PGExplainer, SubgraphX, GraphMask and GraphLIME are left out.

Lavecchia [4], in WIREs Computational Molecular Science, treats SHAP, attention-based visualization, concept whitening and saliency mapping in technical detail, with attention to how their outputs can be read against chemical knowledge. The 2026 Computer Science Review survey by Gangwal and Lavecchia [5] casts the net wider, sorting XAI methods across drug-discovery settings by input modality, model transparency and interpretability objective, and taking in fidelity, stability, sparsity, mechanistic plausibility and task-dependent method selection along the way. None of these criteria is new here, and neither are the explanation methods behind them. What differs is the problem they are organized around. Those surveys take drug discovery as the unit of coverage; this review instead begins with a single network-pharmacology claim made on a heterogeneous biological graph, and asks how that claim ought to be audited.

What separates the two is scope and operational use, not ownership of the underlying criteria. An explanation in graph-based network pharmacology might take the form of a typed subgraph, or of a multi-hop path running from drugs through targets and pathways to phenotypes and diseases. Assessing such an explanation requires attention to the relation direction, edge provenance, graph connectivity, node degree, literature popularity, and possible overlap between training data and the external resource used for comparison. It also requires a clear boundary between model-level fidelity and evidence for a biological mechanism. These graph-specific demands define the question addressed by this review.

The practical contribution is a claim-level audit pathway for that setting. The five domains ask whether an explanation is faithful to the model, stable under reasonable variation, concordant with genuinely external biological knowledge, robust to graph and annotation biases, and linked to an appropriate translational follow-up. The accompanying R1-R6 checklist converts those domains into information that authors can report for an individual network-pharmacology analysis. This framework does not replace method taxonomies or benchmark studies; it connects method choice, graph-specific controls, evidentiary boundaries, and the next testable study in one reporting sequence.

We do not claim to have introduced faithfulness, stability, biological concordance, bias assessment, or translational evaluation as individual concepts. Each is already established in the XAI and graph-learning literature: faithfulness and sparsity metrics are formalized in GNN-explainability evaluation frameworks [6,7], motif-level agreement has been used to benchmark molecular explanations [8], and regulatory guidance already asks for context of use, limitations, and residual uncertainty [9]. Transparency features are likewise present in the systems examined later in this review. TxGNN couples zero-shot repurposing with a dedicated explainer module and clinician-facing rationales [10], DDI-GCN builds interpretability into a graph-convolutional predictor of drug–drug interactions [11], and MINERVA returns an explicit reasoning path by construction [12].

Table 1 makes this boundary explicit. The contribution is integration and operationalization for heterogeneous network pharmacology, not invention of the individual XAI concepts. A broad taxonomy helps researchers understand and select an explanation method. The framework proposed here is intended to help them audit a particular graph-derived claim: why the method fits the task, whether the explanation tracks the model, whether it survives stability and bias checks, what kind of external evidence supports it, and what experiment or clinical analysis would be needed next. The five domains and R1–R6 checklist remain conceptual proposals; they are unweighted, uncalibrated, and have not been empirically validated.

Used in practice, the framework starts from one prediction and its explanation, not from an explanation method considered in the abstract. R1 fixes the task and justifies the method chosen for it. R2 and R3 record fidelity, compactness, stability and uncertainty. R4 tests concordance against a genuinely external source, checking first whether that source overlaps the training data. R5 looks at degree, provenance, novelty and subgroup sensitivity. R6 closes by naming the evidence category reached, the main limitation still outstanding, and the study that should come next. Clearing all six leaves a computational hypothesis that has been audited rigorously; it does not leave a validated biological mechanism.

### 1.4. Scope and Critical Narrative Review Methodology

This article is a critical narrative review rather than a systematic review or meta-analysis. Four sources were searched: PubMed, Web of Science Core Collection, Scopus, and arXiv. The search covered work published between 1 January 2019 and 15 August 2026, which was also the date of the final search. Two families of search terms were combined. The first described the application domain: “network pharmacology,” “polypharmacology,” “drug–target interaction,” “drug repositioning,” “disease–gene association,” “biomedical knowledge graph.” The second described the interpretability problem: “explainable artificial intelligence,” “XAI,” “interpretability,” “explanation,” “feature attribution,” “graph neural network,” “attention,” “counterfactual,” “rule learning,” “path reasoning.” Named methods were searched individually, including SHAP, LIME, Integrated Gradients, GNNExplainer, PGExplainer, SubgraphX, GraphMask, GraphLIME, CF-GNNExplainer, PGM-Explainer, MINERVA, DeepPath, AnyBURL, and Neural LP. A representative string, adapted to the syntax of each database, was: (“network pharmacology” OR “polypharmacology” OR “drug–target interaction” OR “drug repositioning”) AND (“explainable artificial intelligence” OR “XAI” OR “interpretability” OR “explanation”) AND (“graph neural network” OR “knowledge graph” OR “graph embedding”). Foundational work published before 2019 was not retrieved through the date-limited search. It was identified instead by following reference lists of the retrieved articles and by targeted searches on method names, and was retained where it introduced an architecture, metric, or concept still in active use. Reference lists of included articles and of closely related reviews were screened throughout for additional sources.

Records were eligible if they were written in English and met at least one of three criteria: (i) they developed or evaluated an explanation method applicable to graph-structured data; (ii) they applied AI to drug–target, drug–disease, protein-interaction, polypharmacology, or mechanism-of-action tasks; or (iii) they set out evaluation principles, metrics, or regulatory expectations relevant to explanations on biological graphs. Records were excluded if they addressed interpretability only for non-graph data without transferable methodology, reported predictive performance with no explanation component, concerned clinical decision support unrelated to molecular or network-level reasoning, or were editorials, abstracts, or posters without sufficient methodological detail. Peer-reviewed primary studies were prioritized. Reviews were used to position the field and to locate further primary studies rather than as primary evidence. Peer-reviewed conference proceedings were treated as primary sources, since several foundational XAI and graph-learning methods appear only in that form. Preprints were admitted only where a topic was moving faster than the peer-reviewed literature and no equivalent peer-reviewed report existed; they are identified as preprints at the point of citation, and no conclusion in this review rests on a preprint alone. Screening was conducted by the authors on the basis of title, abstract, and, where necessary, full text. Selection was purposive rather than exhaustive: the aim was to assemble a set of sources sufficient to support a methodological comparison across the main families of explanation methods, not to capture every publication in the field. Sources were then grouped by method family, by network-pharmacology task, and by the type of evidence each study offered, and were synthesized critically against the three graph-specific interpretability demands set out in Section 3.1.

The article was conceived and conducted as a critical narrative review. That design was chosen to allow interpretive comparison across a body of work spanning computational architectures, graph structures, prediction tasks, explanation outputs, benchmark datasets, biological evidence types, and regulatory guidance, and to allow conceptual and methodological sources to be read alongside primary studies. A systematic search and selection process would reduce selection bias, and we do not suggest otherwise. The records needed for a fully reproducible retrospective systematic-review workflow, including complete database exports, duplicate-removal logs, and prospectively documented screening decisions, were not retained during the work. Relabeling the article as a systematic review after the fact, or claiming PRISMA compliance, would therefore misrepresent how it was produced. We have instead strengthened the methodological reporting described above and state the resulting limitations below. The design increases susceptibility to selection and interpretation bias. The review was not registered and no prospective protocol was published. Selection was purposive rather than exhaustive, and the article should not be read as an exhaustive synthesis. Screening was not conducted by independent duplicate reviewers, and no inter-rater agreement was calculated. No claim of PRISMA compliance is made. Coverage is limited by the four databases searched and by terminology that varies across the machine-learning and pharmacology literature, where the same idea often appears under different names; relevant work indexed elsewhere, or described in vocabulary we did not anticipate, may have been missed. Restriction to English-language sources introduces a further limitation. Publication bias applies with particular force here, since methods that perform well are more likely to be reported than methods that fail, and negative or null explanation results are rarely published at all. The field is also moving quickly, and work appearing after the final search date is not represented. Quantitative results are reported only when traceable to a cited source. No dataset was analyzed, no model was trained or rerun, and no empirical benchmark was generated for this review. The five-domain framework and the R1–R6 checklist are conceptual contributions that require prospective evaluation, calibration, and consensus development, as set out in Section 9.5.

Appendix A (Table A1) makes the evidence base easier to examine by listing representative primary studies used in our discussion of attribution, subgraph, counterfactual, probabilistic, path-based, and biomedical graph explanations. It is intended as a structured guide to the purposively selected literature, not as a systematic-review flow count or a complete inventory of retrieved studies.

### 1.5. Structure and Scope

We begin by asking what interpretation actually means on a heterogeneous biological graph. From there the review works through the main families of XAI methods, the circumstances in which each tends to fail, and the tasks each suits best. Multi-omics integration, translational evidence, benchmark design and emerging technologies follow. The closing sections draw these threads together into reporting recommendations and priorities for future work.

### 1.6. Central Hypothesis and the Network Pharmacology 2.0 Framework

We use “Network Pharmacology 2.0” as an organizing idea, not as an established classification of the field. Its premise is a simple one: explanation should be designed and evaluated alongside a network-pharmacology model, rather than bolted on after prediction as a visual summary. Five related principles set out what that would involve.

1. Structural interpretability: Where the architecture permits it, the model should expose bounded paths, relation-specific contributions, rules, or some other auditable intermediate representation. Where a post hoc explanation cannot be avoided, its fidelity to the predictor should be measured and not assumed.
2. Multimodal grounding: A prediction drawn from a graph should be set against independent sequence, structural, perturbational or omics evidence. Two models agreeing with each other can be informative, but agreement is not direct evidence in Categories C–G, least of all when the two were trained on overlapping data.
3. Evidentiary discipline: An observational association, an estimate from a causal model, and an effect confirmed by intervention sit at different levels of evidence, and should be labeled as such. Causal language also carries specific reporting obligations: the underlying assumptions should be explicit, and the causal graph and adjustment set should be provided when the analysis requires them.
4. Governed trustworthiness: Where the data allow it, both predictive and explanation performance should be examined by entity novelty, node degree, data source, and clinically relevant subgroup. Such analyses can reveal sensitivity to bias. They do not remove the bias on their own.
5. Translational accountability: A prioritized prediction should lead to a specific experiment or clinical analysis that could support or falsify the proposed mechanism. Explanation can sharpen the design of that evidentiary follow-up step, but it cannot stand in place of it. These five principles are a conceptual organizing device proposed by the present authors. They are working definitions for evaluation, not empirically established regulatory criteria, and they have not been tested empirically.

**Figure 1:**
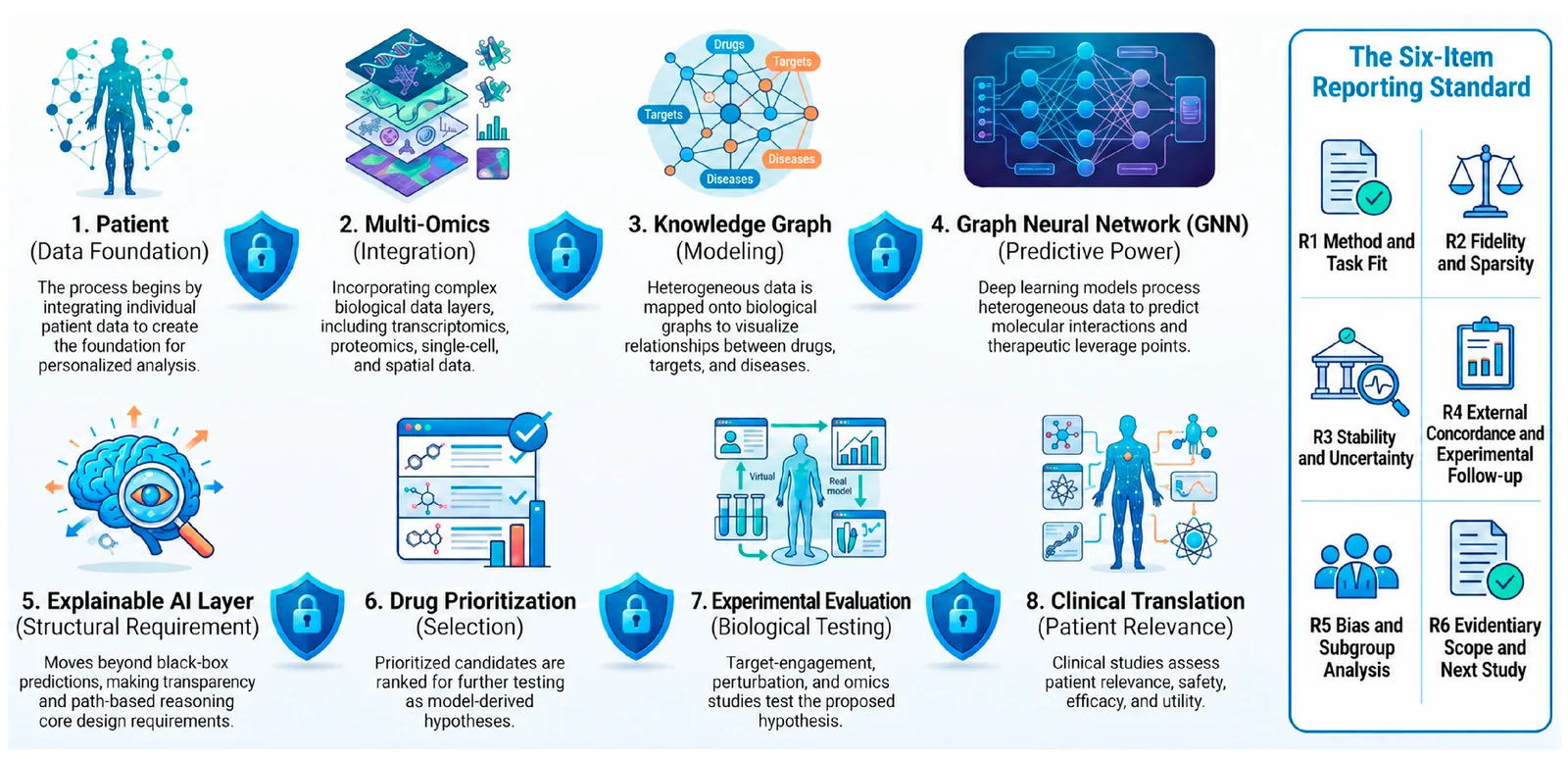
Network Pharmacology 2.0: an explanation-gated workflow. The eight stages run from patient data and multi-omics integration through knowledge-graph construction, graph-model prediction, explainable AI, drug prioritization, experimental evaluation, and clinical translation. The lower panel presents the six-item R1–R6 reporting standard: method and task fit; fidelity and sparsity; stability and uncertainty; external concordance separated from experimental follow-up; bias and subgroup analysis; and evidentiary scope with the next study required. The workflow and reporting standard are conceptual proposals and have not been empirically evaluated or calibrated. Developed by the authors as a synthesis of the network-pharmacology, biomedical knowledge-graph, explainable drug-repurposing, regulatory-AI, and translational literature [1,2,9,10,13,14].

The comparisons of methods and failure modes that follow are organized around these principles, which serve as working definitions for evaluation rather than empirically established regulatory criteria. Figure 1 summarizes the staged framework and indicates where explanation is examined along the pipeline.

## 2. Network Pharmacology Foundations

### 2.1. Core Principles: Polypharmacology, Network Topology, and Disease Modules

Network pharmacology leaves behind Paul Ehrlich’s “one drug, one target, one disease” model. It views the cell as a connected system of genes, proteins, and metabolites in which physiology and disease emerge from interactions rather than a single node. Hopkins articulated the modern form of this view [1], and Boran and Iyengar subsequently developed it through systems pharmacology [15]. Three topological concepts recur. Hubs are highly connected nodes that may influence network stability. Modules are functionally related groups of genes or proteins that may correspond to disease mechanisms. A drug’s topological fingerprint, meaning its position in the network, can also provide clues about intended efficacy and off-target liability.

Network medicine applies the same reasoning to disease. Barabasi, Gulbahce, and Loscalzo described disease as disruption of a network module rather than a defect confined to one gene [16]. On that view, therapeutic relevance may depend on the proximity of drug targets to genes in the affected module. This principle remains central to network-based drug repositioning, linking early protein-interaction overlap studies with newer knowledge-graph embeddings and analyses of potentially synergistic multi-target relationships in oncology [17].

### 2.2. From Manual Network Analysis to AI-Augmented Network Pharmacology

**Figure 2:**
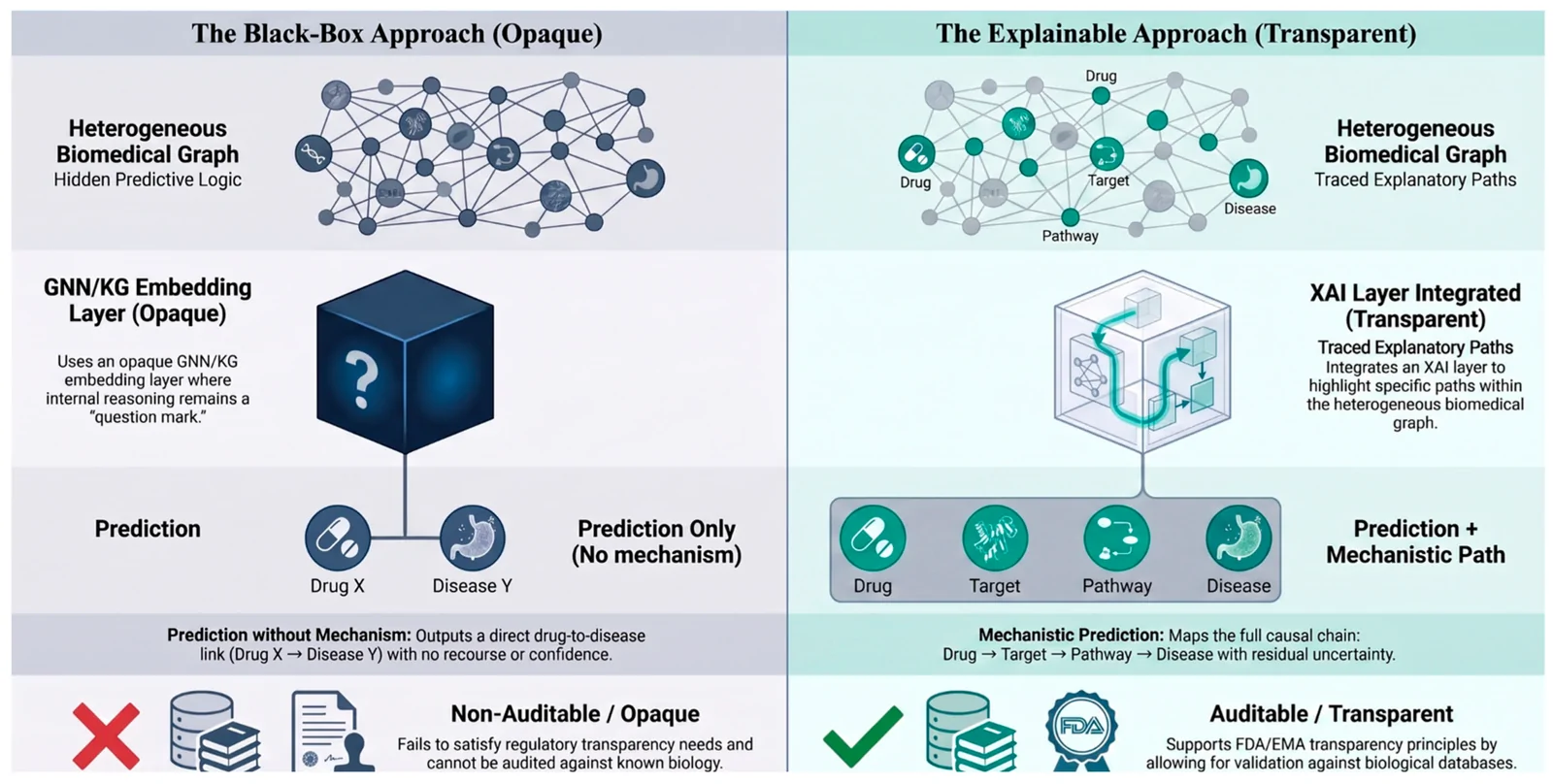
From black-box prediction to an inspectable explanation in network pharmacology. In the black-box configuration, a graph neural network (GNN) or knowledge-graph embedding model converts a heterogeneous biomedical graph into a drug–disease prediction without revealing which relationships influenced the result. In the explainable configuration, an explainable artificial intelligence (XAI) layer identifies the features, edges, subgraph, or drug–target–pathway–disease path used by the model. This output makes the prediction easier to examine and test, but it remains a model-derived hypothesis rather than proof of a biological mechanism. The comparison reflects current XAI, biomedical knowledge-graph, and regulatory-transparency principles [3,4,5,9,13,19].

For roughly the first decade of network pharmacology’s development, these principles were applied through comparatively small, manually curated network pathway databases, disease–gene association lists, and herb–target–disease triads in traditional medicine research analyzed with graph-theoretic metrics (degree, betweenness, modularity) computable without machine learning. The transition to machine learning, deep learning, graph neural networks, and knowledge-graph embeddings over the past several years has been comprehensively traced elsewhere [18], most recently and thoroughly in Li et al.’s [2] review of AI and network biology for polypharmacology in signaling drug design; readers seeking a full historical treatment of that computational evolution are directed there rather than having that ground retraced here. As illustrated in Figure 2, that evolutionary trajectory runs from the traditional and classical roots of the discipline through multi-omics integration and AI-driven prediction to the precision-pharmacology stage.

The methodological consequence of this transition is more important here than its chronology: as network pharmacology moved from graph metrics a human could compute and interpret by hand toward deep models operating on graphs with tens of thousands of nodes and millions of edges, the interpretive transparency that had been, in effect, free in the manual-analysis era had to be actively re-engineered back into the pipeline. The field’s familiar challenges—incomplete and heterogeneous biological data, computational complexity, biological system redundancy—are compounded by a further, less frequently named problem. Predictive models have outpaced the field’s ability to explain their own output in terms compatible with the hub–module–fingerprint vocabulary the discipline was built on.

### 2.3. Why Black-Box AI Is a Specific Bottleneck for Network Pharmacology

This is not a generic complaint about deep learning opacity; it concerns network pharmacology’s founding premise directly. The whole case for treating disease and drug action as network phenomena rests on the mechanism being the object of interest—which hub, which module, which cross-talk pathway—not simply the prediction of a drug–disease or drug–target label. So when a model predicts a repositioning candidate with high accuracy but cannot say whether that prediction rests on genuine proximity to a disease module or on a spurious shortcut (node degree, literature popularity, a batch artifact buried in the knowledge graph), it has, in a real sense, failed at the task network pharmacology actually set out to solve, however good its accuracy metrics look. Figure 2 contrasts this opaque black-box configuration with an explainable alternative in which the drug–target–pathway–disease path underlying a prediction is traced explicitly.

The central interpretability problem is, therefore, what interpretability means, and what it specifically requires, when the object being interpreted is not a flat feature vector or even a single molecular graph, but a heterogeneous, multi-hop, biologically annotated network.

## 3. Interpretability on Biological Graphs

### 3.1. What Makes Network-Pharmacology Interpretability Different?

Most of the XAI literature that SHAP, LIME, Integrated Gradients and saliency maps were developed for, and are still overwhelmingly benchmarked on, consists of either flat feature vectors (tabular QSAR models) or single, homogeneous molecular graphs (one compound, one prediction). Network pharmacology poses a structurally different problem along at least three axes.

Heterogeneous node and edge semantics: A drug–target–disease–pathway knowledge graph contains several node types (such as drugs, proteins, diseases, pathways, and phenotypes) and relations with different meanings and evidentiary strengths. An attribution score is therefore difficult to interpret unless the node type, relation type, direction, and provenance of the supporting edge are reported. Integrated Gradients retains its mathematical completeness property for a differentiable heterogeneous GNN when the baseline and attribution target are well defined [20]. The practical difficulty is biological interpretation: relation-specific transformations make baseline selection and comparison of attributions across relation types non-trivial. Likewise, Shapley-based explanations depend on how coalitions and missing features are defined; conditional and interventional formulations behave differently when graph features are correlated. Relation-stratified attribution and provenance-aware reporting may improve interpretability, but these adaptations require empirical evaluation rather than being assumed mathematically necessary.

Multi-hop, indirect reasoning: Network-pharmacology predictions, particularly drug repositioning and polypharmacology inference, routinely rest on multi-hop paths through the graph (drug → target → pathway → disease) rather than direct edges. This is precisely the “guilt-by-association” logic that gives knowledge-graph embedding its predictive power, but it also means a faithful explanation must surface an entire path, or a small subgraph, rather than a single feature or a single edge weight—a substantially harder attribution problem than flagging important atoms in a molecule.

Biological plausibility as a distinct criterion from statistical fidelity: In image or text classification, an explanation is judged largely by faithfulness to the model. In network pharmacology, an explanation must additionally be judged against independent biological knowledge: does the highlighted subgraph correspond to a known or plausible mechanism? A perfectly faithful explanation that highlights a statistically real but biologically spurious shortcut is faithful and useless simultaneously. This double requirement, statistical fidelity and mechanistic plausibility, is what makes evaluation of network-pharmacology XAI methods a fundamentally harder problem than evaluating XAI on more conventional data types.

### 3.2. A Task-Based Taxonomy of Interpretability Needs

Not every network-pharmacology task requires the same form of explanation. Table 2 maps each task to the type of information an explanation should expose, and to the XAI method families most closely aligned with that requirement.

### 3.3. Trust, Regulatory Expectations, and the Stakes of Getting This Wrong

Interpretability in network pharmacology now sits within a broader regulatory discussion of trustworthy AI. In January 2026, the FDA and EMA published ten guiding principles for good AI practice in drug development, including human-centered design, a clearly defined context of use, data governance, risk-based performance assessment, lifecycle management, and communication of essential information [9]. The principles do not mandate complete mechanistic explainability for every architecture. They instead require information sufficient for users to understand the model’s intended use, limitations, uncertainty, and residual risk. For network-pharmacology applications, this supports reporting an auditable rationale and its limitations rather than presenting a confidence score as a mechanistic explanation.

A network model that recommends a multi-target combination or a repositioning candidate should provide an explanation that a domain expert can examine against available biological evidence. A mechanistic subgraph or path may support that examination, but it remains a model-derived rationale rather than proof of mechanism. Whether such explanations are faithful, stable, and biologically informative remains an open methodological question.

### 3.4. Biomedical Databases and Knowledge Graph Platforms: An XAI-Oriented Comparison

The methods considered here do not operate on data in the abstract; the reliability of any drug–target prediction, path-based explanation, or knowledge-graph embedding depends on the underlying database it draws on. Table 3 compares the databases and knowledge graph platforms most commonly underlying the network pharmacology pipelines used in this field, evaluated specifically from an XAI-relevance perspective; that is, not merely what each resource contains, but what its curation process implies for the bias-exposure checks proposed in this framework.

Critical assessment: resources in Table 3 differ in how they combine experimental, curated, and increasingly text-mined or predicted content within a single database, frequently without distinguishing these provenance tiers in a form downstream GNN or knowledge-graph-embedding pipelines actually consume. This is a direct, upstream contributor to the degree-bias and literature-popularity failure modes described below: a model cannot be expected to distinguish well-established biology from merely well-studied biology if its training data does not make that distinction available in the first place. We recommend that provenance-tier stratification reporting model and explanation performance separately for experimentally derived, text-mined, and purely predicted edges become a standard component of the bias-exposure reporting item in the six-item reporting standard, extending it to a data-provenance check specifically.

### 3.5. Knowledge-Graph Construction and Embedding Platforms

Software infrastructure also constrains which explanations are feasible. Graph databases such as Neo4j support explicit path queries; PyKEEN and DGL-KE provide scalable knowledge-graph embedding workflows; and PyTorch Geometric implements common GNN architectures and explainers. These tools are not directly comparable products: they occupy different layers of the pipeline. Authors should report the software version, graph schema, relation handling, data provenance, and explanation implementation sufficiently to permit reproduction.

## 4. Explainable AI Methods

### 4.1. Framing: Model-Specific vs. Model-Agnostic Interpretability

A first distinction among XAI methods is the source of the explanation. Model-specific approaches use the internal operations of a given architecture, for example gradients in a differentiable network or attention weights in a transformer. Model-agnostic methods such as SHAP and LIME interrogate the model externally, usually by perturbing inputs or fitting a surrogate. That flexibility often entails greater computational cost and may reduce fidelity. The distinction is particularly important in network pharmacology, where heterogeneous GNNs and knowledge-graph embeddings are common even though many model-agnostic methods were developed in quite different settings.

### 4.2. Feature-Attribution Methods

#### 4.2.1. SHAP (SHapley Additive exPlanations)

SHAP is grounded in cooperative game theory. It treats each feature as a player and defines the corresponding Shapley value as the feature’s average marginal contribution across possible player orderings [30]. Its main theoretical attraction is a uniqueness result: among additive attribution methods, SHAP alone satisfies local accuracy, missingness, and consistency simultaneously.

Applications and limitations in network pharmacology: SHAP is often used to rank molecular descriptors, genes, or structural features, but the resulting attribution depends on the background distribution and on whether the expectation is conditional or interventional [30]. In omics and interactome data, correlated variables may exchange importance, making a ranking appear more definitive than the evidence warrants. Sensitivity can be examined by resampling, varying the background set, or using grouped or graph-aware coalitions. GStarX, for example, preserves graph connectivity when it forms coalitions [31], although its behavior on large heterogeneous pharmaceutical knowledge graphs remains insufficiently tested. Exact Shapley values are combinatorial, so applied studies generally rely on approximations and should report the sampling scheme and associated uncertainty.

#### 4.2.2. LIME (Local Interpretable Model-Agnostic Explanations)

LIME takes a different approach: it approximates a complex model’s local behavior around one specific prediction by fitting an interpretable surrogate to perturbed samples drawn from that instance’s neighborhood [32]. For network pharmacology, the weak point is the very idea of “locality” randomly deleting edges to perturb a node’s neighborhood in a biomedical graph, which can generate instances that make no biological sense at all, undermining the surrogate model in a way LIME’s original image and text settings never had to contend with.

#### 4.2.3. Integrated Gradients (IG)

Integrated Gradients takes yet another route for differentiable models: it attributes a prediction to input features by integrating gradients along a straight-line path from a baseline input to the actual input, which satisfies the completeness axiom [20]. Most GNN architectures are differentiable enough for IG to apply, but choosing a baseline has no obvious biological analog for a graph node and different, equally defensible baseline choices can change which nodes IG flags as important.

### 4.3. Attention-Based Explainability

Graph attention networks and attention-augmented GNNs assign weights to neighboring nodes during message passing. Reusing those weights as an explanation is attractive because it requires almost no additional computation. Yet attention is not causal importance. A large weight shows what the message-passing mechanism emphasized; it does not establish what a counterfactual test would identify as decision-relevant. Evidence from a February 2026 evaluation of single-cell foundation models supports this caution: attention largely recovered gene co-expression patterns rather than unique regulatory signals [33]. Attention is therefore best treated as a lead. It can direct scrutiny toward interesting nodes, but it is not, by itself, an experimentally supported biological explanation.

### 4.4. Counterfactual Explanations

Counterfactual explanation addresses a different question from attribution: what is the smallest change to the input that would alter the prediction? In network pharmacology, the answer can motivate a direct experiment. If a protein–protein interaction is truly decisive, for example, removing it should change the downstream prediction. Flat feature attribution does not represent this intervention as naturally. Finding a useful counterfactual is nevertheless difficult because the search for a minimal, sparse, and biologically credible subgraph is combinatorial. Without suitable constraints, optimization may favor graph edits that are statistically efficient but biologically implausible.

### 4.5. Rule-Based and Symbolic Approaches

Rule-based explanations produce decisions that readers can inspect, either from an inherently interpretable model or by distilling a more complex predictor. They fit claims expressed through topology, module membership, or disease–gene proximity particularly well. Their brevity is also a limitation: a compact rule set may omit higher-order interactions captured by a deep graph model. The appropriate balance between transparency and predictive flexibility therefore depends on the task.

### 4.6. Explainable Graph Neural Networks

The five methods below pursue the same goal: a model-specific, post hoc explanation for an already-trained GNN prediction. They reach it by increasingly different routes, from per-instance mask optimization to combinatorial subgraph search to learned surrogate networks. Read together rather than in isolation, they trace the field’s evolving answer to a single question: which part of the graph made the model say what it said?

#### 4.6.1. GNNExplainer

GNNExplainer was the first general, model-agnostic framework for explaining any GNN-based prediction on any graph task [34]. Given a trained GNN and a target prediction, it learns a soft mask over the computation graph’s edges and node features by maximizing the mutual information between the original prediction and the prediction restricted to the masked subgraph. Its strengths are real: model-agnostic, applicable post hoc to any already-trained GNN, and explanatory at the level of a subgraph rather than a single feature. Its limitations on biological graphs are just as real: the optimization is instance-specific and must be rerun for every new prediction computationally prohibitive at the pharmaceutical knowledge-graph scale; the continuous-relaxed mask requires an arbitrary threshold; and the mutual-information objective has no built-in notion of biological plausibility, so GNNExplainer can select statistically important but mechanistically implausible edges.

#### 4.6.2. PGExplainer

PGExplainer replaces GNNExplainer’s per-instance optimization with a learned, parameterized explainer network trained once across a distribution of instances [35]. It is dramatically more scalable as a result, and naturally supports global pattern discovery. But the shared explainer network is itself a learned approximation of what the underlying GNN attends to, introducing a second layer of approximation error. This faithfulness gap may widen specifically on heterogeneous biomedical graphs, where the explainer’s shared parameters must generalize across structurally dissimilar node types.

#### 4.6.3. SubgraphX

SubgraphX reframes explanation as a combinatorial search problem, using Monte Carlo Tree Search and Shapley values to explore and score candidate connected subgraphs [36]. It directly targets the “surface a coherent subgraph” requirement outlined above, but its search scales poorly with node degree, which is a serious problem for biomedical hub nodes. Follow-on work has argued that Shapley values are, by construction, not structure-aware, since permutation-based marginal contribution ignores which coalitions are graph-connected, proposing structure-aware alternatives (GStarX [31]) for exactly this reason.

#### 4.6.4. GraphMask

GraphMask learns a differentiable, per-layer, per-edge binary mask indicating which edges are erasable at each GNN layer without changing the prediction, trained end-to-end across the full dataset [37]. Layer-wise masking distinguishes where in the reasoning process an edge stops mattering, which can be valuable for multi-hop network pharmacology. However, the method was originally developed and evaluated on NLP graph tasks, not biomedical ones, and its assumption of uniform relevant GNN depth across instances fits pharmaceutical knowledge graphs poorly, where relevant path length genuinely varies.

#### 4.6.5. GraphLIME

**Figure 3:**
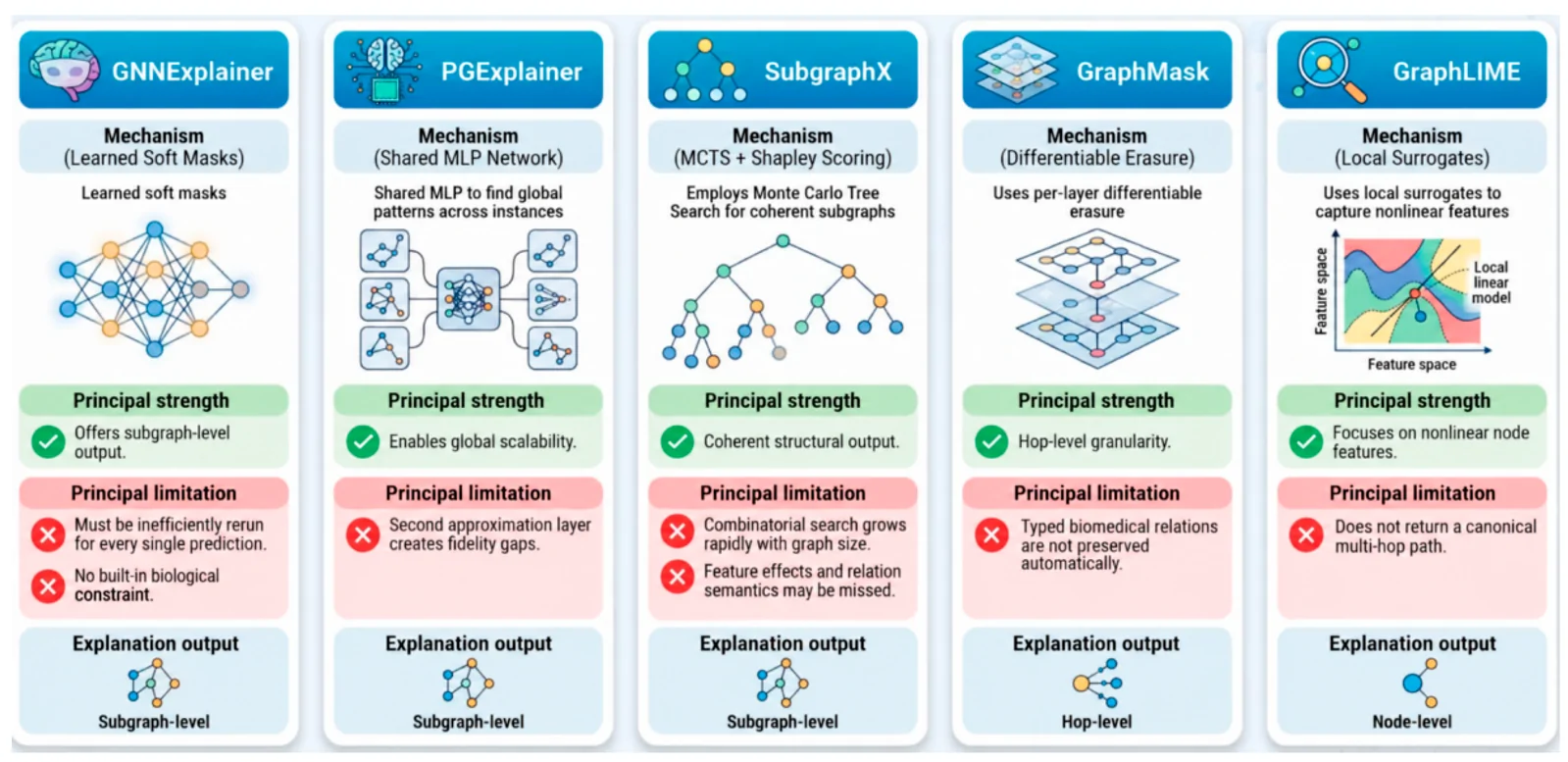
Explainable GNN methods: mechanism, output, and principal caveat. The simplified comparison distinguishes the mechanism, explanation output, and main limitation of GNNExplainer, PGExplainer, SubgraphX, GraphMask, and GraphLIME. These methods return model-level explanations at the feature, edge, hop, node, or subgraph level; none independently establishes a biological mechanism. Detailed strengths, implementation considerations, and biomedical evaluation status are provided in Table 4. Developed by the authors from the original method descriptions [34,35,36,37,38].

GraphLIME extends LIME’s local surrogate-modeling idea to graphs, fitting a nonlinear interpretable model via HSIC-Lasso within a target node’s local subgraph neighborhood [38]. It captures nonlinear feature dependencies LIME’s linear surrogate cannot, and operates on node features as well as topology but it inherits LIME’s “local neighborhood” ambiguity on heterogeneous graphs, and its feature-selection framing explains which features mattered without specifying which path or mechanism connected them to the outcome. These five explainable GNN methods are compared side by side in Figure 3, which contrasts their underlying mechanisms, relative strengths and weaknesses, and the level at which each returns an explanation.

**Table 4:** Comparison of XAI method families across the three interpretability requirements of network pharmacology, including their main computational procedures and the current state of biomedical evaluation.

Method Family	Heterogeneous Semantics	Multi-Hop or Path Output	Built-In Biological Constraint	Computational Procedure and Scaling Consideration	Biomedical Evaluation Status
SHAP/IG	Does not intrinsically preserve typed node or relation semantics	Returns feature attributions, not an explicit multi-hop path	No biological constraint is built into the attribution objective	Requires background sampling or path integration; cost varies with feature count, sample count, and implementation.	Retrospective biomedical applications reported
LIME/GraphLIME	GraphLIME uses a local graph neighborhood, but typed relation semantics are not intrinsic	Returns local feature relevance, not an ordered multi-hop path	No biological constraint is built into the surrogate objective	Requires repeated perturbation and local surrogate fitting for each explained instance.	Limited retrospective biomedical evaluation reported
Attention weights	Typed inputs can be supported by the underlying architecture; relation-specific explanations are not guaranteed	Returns local neighbor weights, not an explicit reasoning path	No; attention weight is not evidence of biological or causal importance	Reuses attention weights produced during inference; no separate explainer optimization is required.	Retrospective biomedical applications reported; explanatory validity remains contested
GNNExplainer	Can accept typed graph inputs, but the learned mask does not inherently preserve relation semantics	Returns an explanatory subgraph, not necessarily an ordered path	No biological constraint is built into the optimization objective	Requires per-instance mask optimization and repeated model evaluation.	Retrospective biomedical applications reported
PGExplainer	Can accept typed inputs; relation semantics depend on the underlying model and implementation	Returns explanatory edge or subgraph scores, not an ordered path	No biological constraint is built into the explainer objective	Requires one explainer-training stage; subsequent explanations reuse the trained generator.	Limited biomedical evaluation identified
SubgraphX	Treatment of typed nodes and relations depends on the implementation	Returns a connected explanatory subgraph, not necessarily an ordered path	No biological constraint is built into the search objective	Uses combinatorial subgraph search; the candidate space grows rapidly with graph and explanation size.	Limited biomedical evaluation identified
GraphMask	Typed relation semantics are not intrinsic to the masking procedure	Returns layer-specific edge masks rather than one ordered path	No biological constraint is built into the masking objective	Requires a separate mask-learning stage; subsequent explanations reuse the learned masking model.	No biomedical evaluation identified in the cited literature
CF-GNNExplainer	Treatment of typed nodes and relations depends on the underlying model	Returns a minimal counterfactual graph edit or subgraph, not an ordered path	No built-in biological constraint; permissible edits can be constrained by the user	Requires a counterfactual graph search for each explained instance.	Limited biomedical evaluation identified
Causal-GNN (OrphicX/GEM)	Support for typed semantics depends on the architecture and implementation	Returns explanatory subgraphs or latent causal factors rather than a standardized ordered path	Uses a causal objective, but this does not constitute biological confirmation	Requires causal decomposition or auxiliary-model training, depending on the implementation.	Limited retrospective biomedical evaluation reported
PGM-Explainer (Bayesian)	Typed relation semantics are not intrinsic to the local Bayesian-network construction	Returns a local probabilistic dependency graph, not an ordered path	Models probabilistic dependence but contains no built-in biological constraint	Requires perturbation sampling, variable selection, and local probabilistic-structure learning.	No biomedical evaluation identified in the cited literature
Path-based KG (MINERVA/DeepPath)	Retains explicit relation types used during graph traversal	Returns an explicit ordered multi-hop reasoning path	No; an inspectable path still requires independent biological assessment	Requires sequential path search; burden depends on path length, branching factor, and search strategy.	Retrospective knowledge-graph evaluation reported; direct biomedical evidence remains limited
Rule mining (AnyBURL/Neural LP)	Retains explicit relation types within learned rules	Returns explicit relational rules or compositional paths	No; learned rules do not establish biological validity	Requires rule induction or differentiable rule learning; scaling depends on graph size and rule-search space.	Limited biomedical evaluation identified

### 4.7. Causal Explainability

Attribution and counterfactual-masking methods explain a model prediction; they do not, by themselves, identify the biological cause of an outcome. CF-GNNExplainer searches for a minimal graph perturbation that changes a GNN prediction [39], and its evaluation showed that factual explainers adapted to the counterfactual task performed less effectively than a method designed for that purpose. Other causal-GNN approaches, including OrphicX and GEM, generate causally informed hypotheses from observational graph data. Without interventional evidence, however, these outputs should not be described as confirmed causal mechanisms. Perturbational experiments, including CRISPR-based studies and Perturb-seq, provide a stronger evidentiary basis for causal interpretation.

### 4.8. Bayesian Explainability

Where the methods above produce point-estimate explanations, Bayesian explainability approaches produce distributions over explanations, surfacing the model’s own confidence in its attribution. PGM-Explainer perturbs a target node’s neighborhood, performs variable selection via the Markov blanket of the perturbed variables, and fits a Bayesian network over the surviving variables through structure learning [40], producing an explanation that is itself a small probabilistic graphical model. This structure maps naturally onto network pharmacology’s own conceptual vocabulary and directly serves the regulatory principle of communicating residual uncertainty. But Markov blanket discovery and Bayesian structure learning are statistically demanding procedures, and they degrade with the small, noisy perturbation samples realistically available around any single node in a large pharmaceutical knowledge graph; this method family has received limited evaluation at the scale pharmaceutical knowledge graphs actually reach.

### 4.9. Interpretable Knowledge-Graph Reasoning

Knowledge-graph reasoning provides a relatively direct route to interpretation. MINERVA [12] and DeepPath [41], for example, move through a graph one relation at a time, leaving a path that can be inspected. In drug repositioning or mechanism-of-action analysis, a drug–target–pathway–disease path offers a concrete mechanistic hypothesis for testing. Symbolic rule learners such as AnyBURL [42] and Neural LP [43] provide a complementary representation. Visibility is not validity, however. An inspectable path may still be biologically wrong, and its performance must be compared with dense embedding methods such as TransE [44], RotatE [45], and ComplEx [46] on the task at hand. Hybrid designs can combine embeddings with path- or rule-based explanations, but the explanatory layer still requires a direct fidelity test.

### 4.10. Synthesis: No Free Lunch Across the Interpretability Frontier

Table 4 consolidates the methods above against the three graph-specific demands, together with each method’s principal computational procedure and the present status of biomedical evaluation.

For comparison, we group the methods into four broad families: feature attribution (SHAP and Integrated Gradients), explainable GNNs (GNNExplainer and SubgraphX), path-based knowledge-graph reasoning (MINERVA and DeepPath), and causal or Bayesian graph explainers (CF-GNNExplainer and PGM-Explainer). Each family returns a different explanatory object, such as features, subgraphs, paths, or probabilistic dependencies. Their performance should therefore be judged with criteria suited to the task rather than as though the outputs were interchangeable.

Table 4 avoids ordinal labels and instead describes what the cited methods report. The heterogeneous-semantics column asks whether an explanation retains node and relation types; the path-output column records the explanatory object produced; and the biological-constraint column notes whether biological constraints are built into the method. The computational-procedure column summarizes the main operations and likely scaling issue, while the final column describes the biomedical evaluation reported in the cited literature. These entries synthesize published evidence; they are not new measurements or a numerical ranking. Computational demand and evaluation status will also vary with the task and implementation.

The comparison is deliberately qualitative, since no harmonized biomedical benchmark with common data splits and explanation metrics yet exists. The ratings represent an evidence-informed synthesis rather than a numerical ranking, and reproducible task-specific comparisons should eventually replace them.

## 5. Failure Modes in Graph-Based Network Pharmacology

### 5.1. Technical Grounding: GCN, GraphSAGE, GAT, GIN

Four GNN architectures recur in biomedical graph learning. Graph Convolutional Networks aggregate neighboring features through normalized graph structure [47]. GraphSAGE enables inductive learning by sampling and aggregating neighborhoods, though the explanation it yields may depend on which computation graph was sampled [48]. Graph Attention Networks learn neighbor-specific weights [49], and those weights are not in themselves evidence of causal importance. Graph Isomorphism Networks are highly expressive for message-passing tasks [50] while their internal representations remain opaque. Graph-convolutional models have additionally captured complex drug combinations and polypharmacy side-effect relationships [51]. Predictive achievement of this kind should not be read as demonstrated explanation quality.

### 5.2. Heterogeneous and Knowledge-Graph GNNs

Pharmaceutical knowledge graphs link drugs, targets, diseases, pathways, and phenotypes through relations that differ in meaning and evidentiary strength. Because many popular GNN explainers were initially assessed on homogeneous or synthetic graphs, their suitability for heterogeneous biomedical graphs cannot be assumed. Edge importance makes the problem clear. A useful explanation should distinguish an experimentally supported relation from a predicted association or a literature-derived co-occurrence; otherwise, evidence of very different quality is presented as though it were equivalent.

### 5.3. Three Concrete Failure Modes

#### 5.3.1. Degree-Bias Shortcut Learning

Biomedical interaction networks are susceptible to degree-related shortcuts because heavily studied drugs and targets tend to accumulate more recorded links. The risk is plausible and testable, but it is not universal. Recent heterogeneous drug–target interaction models discuss imbalance, generalization, and interpretability [52], although Ref. [52] does not itself establish degree bias or the previously claimed decline in cold-target performance. Demonstrating a degree shortcut requires cold-entity testing, degree-matched controls, degree-only baselines, graph randomization, or a suitable combination. If a model fails these controls, its explainer may accurately highlight connectivity while offering little mechanistic insight.

High degree should not automatically be written off as an artifact. Certain hubs, p53 and AKT among them, really are central to cell-survival signaling, so attribution to their connectivity can reflect genuine biology rather than sampling quirks. The problem arises when degree substitutes for the mechanistic signal and keeps predicting well even after that signal is stripped out. Distinguishing the two takes more than simply flagging a hub. Degree-preserving randomization rewires the network while holding each node’s degree fixed, which lets us ask whether the prediction or the attribution collapses under that change. A complementary null model compares the observed attribution against what would be expected across degree-matched random graphs. Together, these tests help separate signal that survives topological controls from attribution that is really just tracking the literature-rich density of the training graph. If both the prediction and its explanation hold up under degree-preserving randomization, a stronger claim of biological plausibility may be warranted under the external-concordance criterion, though independent evidence is still needed to confirm it.

#### 5.3.2. Hub Over-Attribution

Even where a model is not exploiting degree as a shortcut in any direct way, message-passing architectures propagate more information through high-degree hub nodes for the structural reason that more paths run through them. Explanation methods that score importance via message-passing contribution will therefore tend to over-attribute importance to hub nodes, well-studied kinases, or common pathway intermediates even when a lower-degree, more specific node is the true mechanistic driver, producing explanations that are biologically bland rather than biologically discriminating.

#### 5.3.3. Literature-Popularity Bias

Because pharmaceutical knowledge graphs are built substantially from the published, curated literature, well-studied drug classes and disease areas are systematically over-represented relative to their true biological importance. A GNN trained on such a graph can partially reproduce this literature-attention pattern rather than an underlying biological reality, and a faithful explanation of such a model will faithfully report that pattern as the basis for its prediction, since that genuinely is what the model learned. No feature-attribution method can distinguish “this reflects real biology” from “this reflects what has been published about” using the graph data alone. Among these three failure modes, literature-popularity bias is the hardest to correct after the fact, since degree-preserving randomization and message-passing analysis both operate on the graph as given: correcting for it requires external, non-literature-derived evidence and experimental screening data, for instance, rather than any adjustment internal to the graph or the model.

### 5.4. How XAI Methods Address, or Fail to Address, These Failure Modes

Table 5 summarizes how each XAI method family considered above addresses, or fails to address, the three failure modes identified above.

None of the XAI methods considered here reliably detects these three failure modes; at best, they make the failure mode visible for human scrutiny rather than automatically correcting for it. Explainability, as currently practiced, is a diagnostic aid requiring an informed human in the loop, not an automated safeguard. Mitigating these biases requires action upstream of explanation entirely, debiased graph construction, degree-stratified evaluation, time-split or leave-one-source-out benchmarking mitigations developed fully as reporting requirements in the evaluation framework.

## 6. Applying XAI Across the Network-Pharmacology Pipeline

Across the applications considered below, supporting evidence is generally more developed for single-molecule and single-endpoint explanations than for heterogeneous, multi-hop network explanations. This pattern should be interpreted as a difference in the amount and type of available evidence, not as a universal inverse relationship between network scale and XAI quality.

### 6.1. Drug–Target and Protein–Protein Interaction Prediction

Explainable graph models are comparatively mature for molecular interaction tasks, although evidence should not be transferred across tasks without qualification. DDI-GCN applies an explainable graph-convolutional architecture to drug–drug interaction prediction [11]; it is not direct evidence of experimentally supported drug–target or toxicity explanations. More generally, a substructure- or edge-level explanation is informative only when the underlying model has been evaluated on genuinely novel entities and the highlighted features have been assessed independently.

### 6.2. Drug Repositioning and Polypharmacology

Drug repositioning is a natural setting for path- and subgraph-based explanation because a prediction can be linked to intermediate targets, pathways, and disease concepts. TxGNN was evaluated under zero-shot disease splits and its Explainer produced multi-hop paths that were assessed through a small human study and comparison with medical evidence [10]. These results support interpretive usefulness, but they do not constitute prospective experimental confirmation of the predicted mechanisms. An automated knowledge-graph reasoning approach has separately reported experimental testing of selected evidence paths, with results consistent with the model-derived hypotheses for the paths examined [53]. That provides Category C and/or Category D evidence for the specific paths examined and does not establish the general reliability of the method. Such examples are encouraging, yet standardized comparisons of explanation faithfulness, stability, and biological validity remain limited.

### 6.3. Network Construction, Clustering, and Pathway Enrichment

XAI’s role here is underdeveloped, in part because the underlying task does not create much of a black-box problem to begin with: clustering and pathway enrichment traditionally rely on statistical overlap tests rather than learned models. The real opportunity is narrower: explaining learned network construction methods, where a GNN or embedding model infers edges not present in curated databases such as Open Targets [28] or STRING [25]. Rigorously evaluated applications specifically for this sub-task remain sparse relative to drug–target and repositioning applications, a real if narrower research gap.

### 6.4. Biomarker and Target Prioritization

SHAP-based feature attribution dominates this space, ranking candidate genes or proteins by their contribution to a disease-classification or drug-response model. Integrated Gradients and DeepLIFT-style gradient attribution have seen similar use in omics-driven research to pinpoint genes influencing drug-response classification. This task is more exposed than most to the hub-over-attribution and literature-popularity failure modes described above, since highly studied genes are structurally likely to receive high attribution regardless of their true disease-specific relevance. Target-prioritization XAI outputs should therefore be reported alongside a degree- or annotation-density control, a check that is rarely reported in the current literature.

### 6.5. Mechanism-of-Action Inference

Mechanism-of-action inference is the task that most directly requires full-subgraph or full-path explanation output. Integrated Gradients applied across multiple distinct preclinical endpoints, hERG channel inhibition, plasma protein binding, passive permeability, and cytochrome P450 inhibition within a single GNN framework shifted its explanatory focus in chemically sensible ways across endpoints, offering qualitative but informative evidence of endpoint-appropriate mechanism tracking. The example is instructive precisely because it operates at the single-molecule structural scale; extending this endpoint-sensitivity finding to network-level mechanism-of-action inference remains an unproven extrapolation.

### 6.6. Toxicity, ADMET, and Clinical Decision Support

Toxicity and ADMET prediction are among the better-developed areas of XAI-enabled drug discovery, although most applications operate at the single-molecule rather than the network level [3]. Feature-attribution methods can identify molecular fragments associated with a model output, but these patterns remain model explanations rather than experimentally confirmed toxicological mechanisms. For decisions with regulatory implications, explanation should be accompanied by the model’s context of use, known limitations, performance uncertainty, and an appropriate evidentiary strategy [9].

### 6.7. Synthesis Across the Pipeline

Available evidence suggests that explanation methods are more extensively evaluated for single-molecule and single-endpoint tasks than for heterogeneous, multi-hop network applications. This uneven maturity motivates the proposed evaluation framework.

## 7. Multi-Omics and Emerging Data Integration

### 7.1. XAI for Multi-Omics-Integrated Network Models

Network-pharmacology models increasingly place transcriptomic, proteomic, single-cell, and spatial measurements alongside drug–target–disease graphs. This can sharpen biological resolution. It also creates new routes through which technical variation, cell composition, or annotation density may influence a prediction. In a recent assessment of single-cell foundation-model interpretability, attention weights largely recovered broad gene co-expression structure rather than unique regulatory mechanisms [33]. A coherent attribution, then, need not be a mechanistic one. Multi-omics studies should test whether explanatory features persist across preprocessing choices and whether they agree with held-out assays or independent cohorts.

### 7.2. Large Language Models and Foundation Models in Network Pharmacology Reasoning

Foundation models now cover biomolecular structure, biological sequence, single-cell measurements, and biomedical language. AlphaFold 3 and ESMFold model protein structure at different scales and with different formulations [54,55]. Evo operates across molecular and genomic sequence contexts [56], whereas scGPT and Geneformer are single-cell foundation models trained on transcriptomic profiles rather than on drug–target graphs [57,58]. BioGPT, Med-PaLM, and BioMedLM address biomedical or clinical language [59,60,61]. These systems solve different problems; none provides an empirically supported explanation for a network-pharmacology prediction by default. Once their representations enter a biological graph, attribution should separate information inherited from the foundation model from evidence propagated across graph edges. The rationale should also be tested under changes in prompts, embeddings, graph construction, and external evidence.

### 7.3. Digital Twins and Autonomous AI: A Bounded Note

Digital twins and autonomous AI-driven discovery platforms receive only brief treatment here, since Li et al. [2] already cover them comprehensively. Any digital twin or autonomous platform issuing network-pharmacology-informed recommendations inherits every interpretability limitation described above the moment its underlying network model generates that recommendation. Trust in an autonomous system, in other words, is inherited from the explainability of the network-pharmacology models embedded within rather than being engineered independently at the platform level.

## 8. Clinical Translation and Trust

### 8.1. Regulatory Frameworks for Explainability

**Figure 4:**
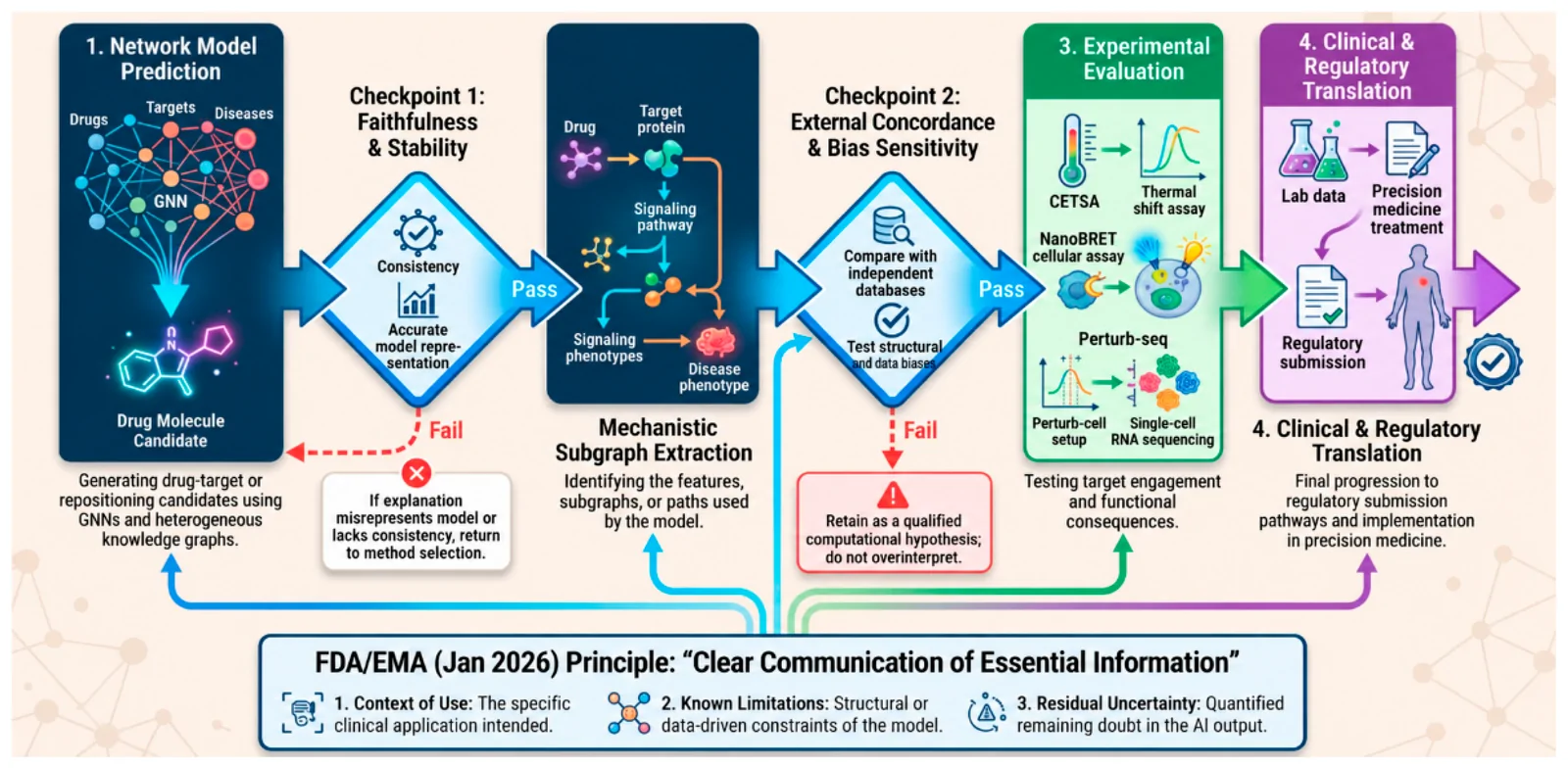
From model prediction to experimentally supported evidence. A model-derived feature, subgraph, or path first passes a model-level gate based on faithfulness and stability. External biological concordance and bias sensitivity then qualify whether the resulting hypothesis is independent of training overlap and structural shortcuts. Category B concordance remains distinct from biochemical, cell-based, animal, and human evidence in Categories C–G. CETSA, NanoBRET, perturbation experiments, and omics readouts can test target engagement or functional consequences, while clinical and regulatory evaluation addresses patient relevance, safety, and utility. Developed by the authors from responsible-AI guidance and the experimental target-evaluation literature [9,10,53,62,63,64].

For network pharmacology, a particularly relevant FDA/EMA principle is the communication of clear, essential information [9]. The guidance does not require one universal explanation format; instead, it emphasizes context of use, limitations, uncertainty, and information needed for competent human oversight. Figure 4 summarizes a proposed translational workflow in which model explanations are examined before experimental and clinical evaluation. The figure represents an author-proposed framework and should not be interpreted as a regulatory requirement.

### 8.2. Case Studies in Which XAI-Derived Hypotheses Were Subsequently Tested Experimentally

Evidence should be separated by the Categories A–G defined in Section 9.2.5. TxGNN generated multi-hop explanatory paths that were evaluated through a small human study and comparison with medical evidence [10]; this supports interpretive usefulness but is not prospective wet-lab confirmation. Pharmacophore-consistent attribution similarly represents retrospective biological concordance. By contrast, automated knowledge-graph evidence generation has been linked to direct experimental support (Categories C–E, depending on study design) in a primary study [53]. Prospective confirmation of network-level mechanisms generated specifically by subgraph or causal-GNN explainers remains uncommon. Accordingly, Category A explanation, Category B external concordance, Categories C–E experimental evidence, and Categories F–G human evidence should not be presented as interchangeable outcomes.

### 8.3. Gaps Between Computational Explanation and Clinical/Mechanistic Proof

An XAI output should be treated as a computational hypothesis, not as a demonstrated mechanism. Its value is nonetheless practical: it makes a prediction open to inspection and helps researchers choose the next experiment. Experimental confirmation and clinical evaluation are still required.

The experiment should be chosen to match the claim. CETSA or NanoBRET can assess whether a compound engages a proposed target in cells [62,63]. CRISPR perturbation or Perturb-seq can examine whether altering a prioritized gene produces the predicted pathway or cell-state response [64]. Independent transcriptomic, proteomic, or metabolomic measurements can test whether the wider network response is reproduced. Because these assays answer different questions, they should not be combined under a single claim of “validation.” Evidence of target engagement, for instance, does not by itself establish a complete multi-hop mechanism or clinical benefit.

## 9. Benchmarking and the Proposed Evaluation Framework

### 9.1. Why General-Purpose XAI Evaluation Is Necessary but Insufficient Here

Current GNN-XAI frameworks quantify fidelity, sparsity, and related properties [6,7], while molecular benchmarks can compare attributions with recognized structural motifs [8]. These evaluations are necessary but insufficient for network pharmacology. Synthetic node-classification graphs provide convenient ground-truth motifs, and single-molecule graphs generally contain relatively uniform node and edge types. Biomedical knowledge graphs instead combine relation semantics, uneven provenance, literature-driven degree distributions, and multi-hop mechanisms for which there may be no single correct explanatory subgraph.

### 9.2. Proposed Evaluation Dimensions

**Figure 5:**
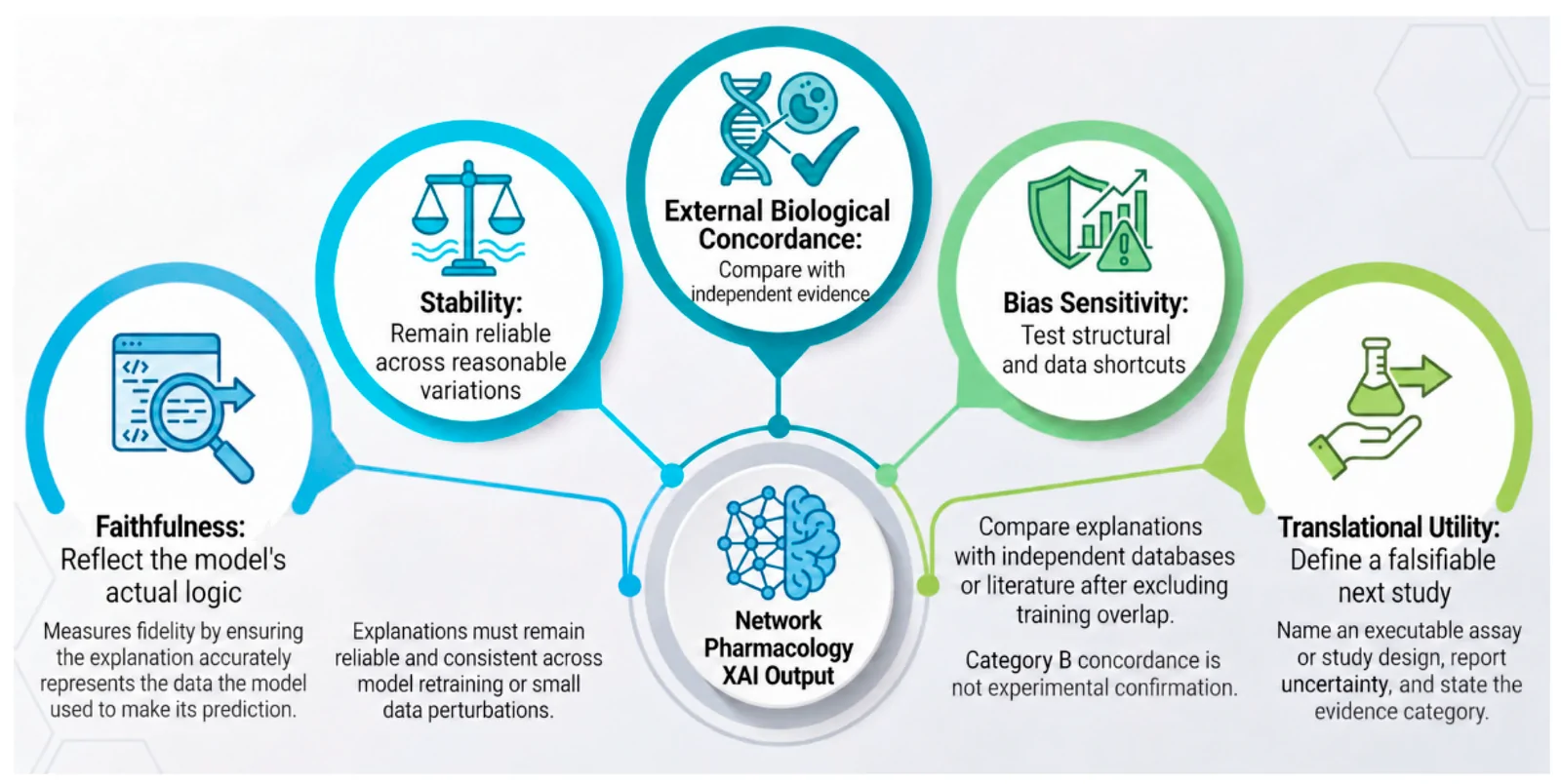
Five domains for evaluating explanations on biological graphs. The same terms are used throughout the manuscript: faithfulness, stability, external biological concordance, bias sensitivity, and translational utility. For each domain, the figure gives an operational question and representative measures. The domains are complementary but have not been weighted or calibrated. Satisfying all five checks produces a carefully audited computational hypothesis, not a biologically validated mechanism. The framework was developed by the authors from established XAI principles, regulatory considerations, and studies of GNN-explanation evaluation [3,4,5,6,7,8,9].

The five evaluation domains used in this review are summarized in Figure 5.

The proposed framework comprises five complementary domains. Faithfulness and stability come from established XAI practice. External biological concordance, bias sensitivity, and translational utility address the additional questions that arise when a computational explanation is interpreted in biological terms.

(1) Faithfulness asks whether an explanation identifies the features or subgraph that actually influenced the predictor. Let f denote the model, G the complete graph, and G_expl the explanatory subgraph. Deletion and retention analyses compare f(G) with *f*(*G*\*G*_{*expl*}_) and *f*(*G*_{*expl*}_) respectively. A deletion measure records how the output changes after removing the explanatory subgraph, whereas a retention measure records the output when that subgraph is kept in isolation. A large deletion effect suggests that the selected elements were necessary; a retained output close to the original suggests that they were sufficient. There is no universally accepted equation. Implementations may use probability, logit, or class output; may report signed or absolute differences; and may reverse the direction in which a higher value is preferable. Even Fidelity+ and Fidelity- are used with inconsistent sign conventions [6,7], so every study should define the quantity it reports. Authors should also specify the perturbation unit, deletion and retention procedures, and the value used to replace removed elements. Zero masking, learned baselines, feature means, and degree-matched substitutes can produce different results on the same graph. Aggregation across instances should be described, preferably with a measure of dispersion. Above all, faithfulness concerns the model’s reasoning. It does not establish that the same reasoning is biologically correct or clinically relevant.
(2) Stability asks whether an explanation persists after retraining, resampling, or a small change to the graph. Agreement between ranked node or edge scores can be summarized with Spearman correlation or Kendall’s tau. For set-valued outputs such as selected subgraphs, Jaccard or top-k overlap is more suitable, provided k is defined in advance. Three perturbation regimes should be kept separate: retraining with different random seeds to examine optimization variance; bootstrap or other resampling of the training data to examine sampling variance; and small structural changes, such as removing a low proportion of edges, to probe sensitivity to graph noise. Studies should justify the number of seeds or resamples and report the distribution and uncertainty of the resulting estimates. We do not propose universal thresholds because acceptable stability depends on the task, graph, explanation size, and consequences of use. A full distribution across runs is more informative than one summary statistic. If rankings vary sharply between seeds, optimization noise may be shaping the result, and hypotheses based on those rankings should be interpreted cautiously.
(3) External biological concordance asks whether the explanation overlaps evidence not used to train the model. A simple overlap fraction may be reported as a descriptive measure, but it should not be called biological plausibility: it is sensitive to reference-database coverage, node degree, provenance, and literature popularity. Concordance should therefore be evaluated against more than one external source, with semantic edge matching, disclosure of training-source overlap, and degree-matched null comparisons. Direct experimental evidence in Categories C–E provides a stronger test of mechanism than Category B database concordance.
  External concordance should be assessed against resources such as Reactome and STRING only after excluding any records used to construct or train the model. Agreement should be stratified by evidence provenance and compared with degree-matched null expectations, because overlap with a literature-rich database can otherwise reproduce the same popularity bias that the evaluation is intended to detect. Operationally, four requirements follow. Any record used to construct, train, or tune the model must be excluded from the comparison set, and the exclusion procedure must be described; without this step the analysis measures memorization rather than concordance. Node and relation matching must be semantic rather than string-based, using explicit identifier mappings, and the treatment of one-to-many mappings should be stated. Concordance should be computed against more than one external resource, since coverage differs systematically between resources. Expected overlap should be estimated from degree-matched random subgraphs rather than assumed to be zero. Two limitations bound the interpretation. External resources are incomplete, so absence of overlap is weak evidence against an explanation. They are also built substantially from the published literature, so agreement is partly a measure of how well studied an entity is. Concordance with Reactome, STRING, or any comparable resource is therefore Category B database concordance, not direct evidence in Categories C–G, and should not be described as confirming a mechanism.
(4) Bias sensitivity examines whether predictions and explanations vary across strata defined by node degree, annotation density, data source, time, or entity novelty. Two kinds of test are useful. First, stratified reporting presents prediction and explanation performance separately by degree band, annotation density, edge provenance, database-entry time, and cold-start novelty. Second, controlled comparisons test whether performance remains after a suspected shortcut is removed. A degree-only baseline shows what connectivity alone can achieve; degree-preserving randomization changes graph structure while retaining the degree sequence; matched-degree controls compare similarly connected entities; and targeted ablation removes a chosen feature or relation type. Differences in fidelity across degree strata show unequal explanatory performance, but they do not alone prove shortcut learning because some hubs are genuinely important biologically. Stronger evidence arises when performance survives removal of the mechanistic signal or when a degree-only baseline approaches the full model. Reports should define the stratification variable, stratum boundaries, number of randomization runs, and comparison statistic.
(5) Translational utility is assessed with a proposed four-item reporting checklist: the explanation should identify a falsifiable next step, name an executable assay or study design, report uncertainty, and state the evidentiary level of the claim. This checklist is an unvalidated reporting aid and should not be interpreted as a clinical actionability score.

An experimentally actionable claim requires an assay matched to the explanation. CETSA can test predicted drug–target engagement in cells, and Perturb-seq can ask whether perturbing prioritized genes produces the expected pathway or cell-state response. Either outcome may strengthen or undermine the model-derived hypothesis. Neither converts an observational graph explanation into causal proof after the fact.

A unified biomedical XAI benchmark is still missing. General-purpose suites can compare explainers on synthetic motifs, but those results should not be used as evidence of biological validity. Conversely, TDC [65], DAVIS [66], KIBA [67], BioSNAP [68], and PrimeKG [13] provide realistic biomedical prediction tasks without a shared explanatory ground truth. A useful field-wide benchmark would combine heterogeneous node and relation types, provenance-labeled edges, temporal and cold-entity splits, experimentally supported mechanism subsets, and standardized fidelity, stability, sparsity, bias-sensitivity, and external-concordance metrics. Until such a resource exists, performance on synthetic graphs and performance on biomedical prediction tasks should be reported as complementary but non-interchangeable evidence.

#### 9.2.1. Benchmark Datasets for Evaluating Network-Pharmacology XAI Claims

Benchmark datasets determine which parts of the framework can be tested. TDC [65], DAVIS [66], KIBA [67], BioSNAP [68], and PrimeKG-derived tasks [13] provide biologically relevant prediction settings, including scaffold, temporal, and cold-entity splits (Table 6). They do not, however, supply a common ground-truth explanation or a harmonized protocol for comparing heterogeneous-graph explainers.

#### 9.2.2. Explainability Evaluation Metrics

Table 7 summarizes established explanation metrics and the proposed biomedical extensions. Metric definitions, perturbation rules, and aggregation procedures should be reported explicitly so that results can be reproduced.

#### 9.2.3. Proposed Operational Criteria for Subgraph Explanations

Subgraph explanations require more than scalar-attribution metrics. We propose that reports address the following points, while recognizing that suitable thresholds remain uncalibrated. Fidelity and sparsity should be presented together because fidelity can be increased trivially by allowing the explanation to expand toward the full computation graph. Sparsity should be given as the retained fraction of nodes or edges, with the denominator made explicit. Sufficiency and necessity correspond to the retention- and deletion-based measures described above and are most informative when reported as a pair. One useful implementation check is to mask all eligible nodes or edges and confirm, within numerical tolerance, that the model returns the output expected for the stated baseline. If it does not, the masking operator, replacement values, residual routes such as self-loops or bias terms, graph preprocessing, or the baseline itself may be introducing an artifact.

Structural criteria matter specifically on biomedical graphs. Connectivity should be checked, since a set of disconnected high-scoring edges does not constitute a mechanistic account. Relation-type preservation asks whether the semantics of each retained edge are reported rather than collapsed into an untyped adjacency. Path validity asks whether a returned path is traversable in the direction implied by the relation semantics, so that a drug-to-disease narrative is not assembled from edges whose directions do not compose. Semantic coherence asks whether the entity and relation types along a path form a biologically interpretable sequence. Provenance should accompany every retained edge, distinguishing experimentally supported relations from predicted or text-mined ones.

Comparison against degree-matched null subgraphs is proposed as a routine control: a subgraph should be shown to score better than subgraphs of comparable size and degree profile sampled from the same graph. Agreement with ground-truth motifs can be reported where such motifs genuinely exist, as in molecular property benchmarks [8], but this is rarely available on heterogeneous knowledge graphs. That limitation is structural rather than incidental. For most biomedical predictions there is no single correct explanatory subgraph, because several redundant or partially overlapping mechanistic routes may each be consistent with current knowledge, and the underlying biology may itself be incompletely characterized. Subgraph evaluation should therefore be treated as a comparison of plausibility and internal consistency, not as scoring against a known answer.

#### 9.2.4. Proposed Handling of Conflicting Criteria

The five domains may point in different directions because they answer different questions. Model-level statistical fidelity asks whether an explanation accurately represents the predictor’s decision process. Mechanistic biological validity asks whether evidence outside the model supports the proposed relationship. An explanation can therefore be faithful yet dominated by high-degree nodes, or concordant with a Category B database while unstable across retraining seeds. We recommend an ordered qualitative assessment rather than a composite score. Even when every conceptual check is passed, the result is a rigorously audited computational hypothesis, not a validated biological mechanism. A single score would require untested weights and could allow strength in one domain to hide a critical weakness in another.

The proposed order runs as follows. Model-level acceptability comes first: unless an explanation shows adequate faithfulness and reproducible stability, it does not reliably describe the model, and no further interpretation is warranted. Bias qualification comes second: where performance depends substantially on node degree, annotation density, edge provenance, or literature popularity, the explanation should carry an explicit bias warning, and any biological reading of it should be downgraded accordingly. Biological support comes third, through provenance-aware external concordance assessed after exclusion of training sources. Translational readiness comes last and requires a falsifiable next step together with evidence appropriate to the strength of the claim.

Several constraints follow from that ordering. Faithfulness is necessary but not sufficient: a faithful explanation of a model that has learned a structural shortcut is an accurate description of the shortcut. External biological concordance does not rescue poor faithfulness, since agreement with a database may reflect the popularity of an entity rather than the reasoning of the model. Translational utility cannot compensate for an explanation demonstrated to be unfaithful, because the proposed experiment would then be testing something the model did not use. Absence of experimental evidence limits the evidence category that may be claimed, as set out in Section 9.2.5, but does not by itself invalidate a model-level explanation, which may remain a legitimate hypothesis-generating output. No configuration of computational or Category B results should be labeled a validated biological mechanism; that claim requires direct evidence appropriate to Categories C–G.

This ordering is a proposal. The sequence reflects our reading of where the risk of over-interpretation is greatest, not an empirically derived rule, and the thresholds separating acceptable from unacceptable performance in each domain remain uncalibrated. Establishing them will require empirical calibration and consensus among methodologists, experimentalists, and regulatory stakeholders.

#### 9.2.5. Proposed Evidence Categories

Much of the ambiguity in this literature comes from treating different kinds of support as interchangeable. We propose that claims arising from explainable network pharmacology state which category of evidence supports them. Category A is a computational prediction or model-derived explanation. Category B is concordance with an external database or the published literature. Category C is in vitro biochemical or molecular evidence, such as a binding or thermal-shift measurement. Category D is cell-based functional or perturbational evidence, such as a perturbation experiment with a measured pathway or cell-state readout. Category E is in vivo animal evidence. Category F is human observational evidence. Category G is human interventional or clinical-trial evidence. These are categories of evidence type and translational proximity, not a ranking of methodological quality or causal strength. A well-designed cell-based experiment may be more informative than a poorly controlled animal study, and the categories should not be read as ordering studies by rigor.

Categories A and B are easily confused and therefore need to be separated explicitly. If a model-derived subgraph agrees with Reactome, STRING, or another curated resource, it is concordant with existing knowledge; this is not experimental confirmation, and calling it validation would overstate the evidence. The remaining categories answer different questions. Biochemical assays examine molecular interaction, cell-based assays examine functional consequences in cells, animal studies examine whole-organism effects, and human studies examine relevance to patients. Observational evidence in people establishes association rather than causality. No category alone resolves mechanism, efficacy, safety, and clinical utility. The evidence required depends on the claim: target engagement and clinical benefit, for example, demand different studies. We apply this terminology throughout the framework, the R1-R6 checklist, the assessment in Section 9.4, and the Conclusions. Item R6 asks authors to state which categories have been reached and what study would move the claim further.

### 9.3. A Proposed Reporting Standard

We recommend that papers applying XAI methods to network-pharmacology tasks report, at minimum:

R1: Algorithmic justification: identify the XAI method and justify its suitability for the prediction task and graph structure, using Table 2 where relevant.

R2: Fidelity metrics: report Fidelity+ and Fidelity−, or another established faithfulness protocol, with the perturbation rule and sign convention defined.

R3: Stability audits: quantify explanation consistency across retraining seeds, resampled data, and small graph perturbations.

R4: External biological concordance and experimental follow-up: report these separately rather than together. For concordance, state the independent databases or literature sources used, such as Reactome or STRING, and the procedure for excluding records used in model construction or training; concordance with a curated resource is Category B agreement with recorded knowledge, not direct evidence in Categories C–G. For experimental follow-up, identify the level of testing performed or proposed, distinguishing biochemical or molecular assays such as CETSA, cell-based functional assays such as Perturb-seq, in vivo animal studies, human observational evidence, and human interventional evidence.

R5: Bias disclosures: report tests for degree bias, hub over-attribution, literature-popularity bias, and other task-relevant data-source effects.

R6: Evidentiary scope: state what the explanation establishes and what it does not, identify which evidence categories in Section 9.2.5 support the claim, indicate whether the supporting evidence is observational or interventional, and name the study or experiment needed to extend the claim.

None of the representative applications examined here reported all six items together. This observation reflects incomplete reporting against the proposed standard, not a claim that the original studies failed their stated objectives.

### 9.4. Evaluation of Representative Pipelines Against the Proposed Framework

Table 8 is an exploratory, literature-based application of the proposed criteria to three systems that differ markedly in purpose and design: TxGNN, a biomedical model for zero-shot drug repurposing with a dedicated explainer [10]; DDI-GCN, an explainable predictor of drug–drug interactions [11]; and MINERVA, a general path-reasoning architecture for knowledge graphs [12]. Its purpose is to demonstrate how the criteria could be applied, not to establish them empirically and not to rank the three systems. Several qualifications govern how the table should be read. The three systems were designed for different objectives and operate on different data structures, so this is not a head-to-head benchmark. MINERVA in particular was developed as a general knowledge-graph reasoning method and not as a biomedical network-pharmacology framework, and several biomedical criteria fall outside the scope its authors set for it. All three publications predate the criteria proposed here, so none could have reported against them. Based only on information available in the primary publications, we classified each criterion as reported, partially addressed according to the proposed criteria, not evaluated in the original study, or not applicable to the study’s original scope. These classifications describe coverage of the proposed criteria alone. They are not assessments of the quality, validity, or importance of the original studies, and an entry of not reported should not be read as a methodological shortcoming.

### 9.5. A Proposed Roadmap for Empirical Evaluation and Calibration of the Framework

Nothing in Section 9.2, Section 9.3 and Section 9.4 constitutes empirical evaluation or calibration of the framework. The domains, the R1–R6 checklist, and the ordering rule are proposals, and the assessment in Table 8 is a demonstration of their use. Setting out what empirical evaluation and calibration would require is therefore part of the proposal itself, and we describe the following as work still to be done rather than work performed here.

Content and construct validity would be established first. An independent multidisciplinary panel, including computational methodologists, medicinal chemists, systems pharmacologists, experimental biologists, and regulatory scientists, would review whether the five domains cover the relevant aspects of explanation quality and whether any domain is redundant or missing. Construct validity would be examined by testing whether domain scores relate to other measures as theory predicts, with convergent validity assessed against established GNN-explainability metrics [6,7,8] and discriminant validity assessed against predictive accuracy, which should not substitute for explanation quality.

The next step would be to determine whether the framework can be applied reliably. This would require written scoring instructions and an assessor manual that defines the evidence needed for each criterion. Pilot use on a small set of publications could then identify criteria that different assessors interpret inconsistently. Agreement should be quantified with statistics suited to the design: Cohen’s kappa, or weighted kappa for ordered categories, for two raters; Fleiss’ kappa or Krippendorff’s alpha for more than two; and an intraclass correlation coefficient if continuous scores replace categories. We do not set target values in advance because acceptable agreement depends on the number of categories and the intended use.

Evaluation should then move beyond the material used to develop the framework. Prospective testing across previously unseen datasets, tasks, and model architectures, as well as different diseases, graph types, data sources, and institutions, would show how the framework behaves outside a single setting. Sensitivity analyses should also examine whether classifications change when alternative fidelity definitions, perturbation strategies, stratification boundaries, or external reference resources are used.

Calibration against meaningful outcomes is the most demanding step. Domain classifications would need to be compared with independent experimental or translational results to determine whether highly rated explanations receive subsequent support more often than poorly rated ones. The strongest test would be prospective: do experiments guided by explanations identify targets or mechanisms more efficiently than prediction alone? Until such evidence is available, the framework should be used as a structured reporting aid and a basis for comparison, not as a calibrated instrument or certification scheme.

## 10. Adjacent Methodological Territories

A number of emerging technologies influence explainability without themselves counting as XAI methods. Protein, genomic, and single-cell foundation models can supply sequence-, structure-, or state-based signals for comparison with graph predictions [54,55,56,57,58]. Such agreement is useful only after training-data overlap and shared literature sources have been examined. Combining two opaque models does not produce a mechanism. Single-cell evidence adds another warning: attention may encode biologically structured but functionally redundant signals [33].

Biomedical language models can speed up the extraction of candidate drug–target and gene–disease relations [59,60,61]. Their generated narratives are not mechanistic evidence, however, and dependence on published text can intensify literature-popularity bias. LLM-based explanations should therefore point back to auditable graph paths and verifiable sources. They should also remain analytically separate from tests of model faithfulness.

Digital twins and federated learning are deployment settings, and both inherit the explanatory limits of their component models. A digital twin requires explanations that are repeated, patient-specific, and timely [14]. In a federated model, an aggregate explanation may not represent any one participating institution. These are worthwhile research problems. They are not evidence that either setting is ready for clinical use.

Regulatory guidance focuses on the context of use, data governance, risk-based performance assessment, lifecycle management, and communication of essential information [9]. It neither endorses a particular XAI method nor requires one universal explanation format. Claims of regulatory alignment should therefore be precise. Without a defined context of use and supporting evidence, network-pharmacology studies should not imply compliance.

## 11. Future Perspectives

In the near term, the field is likely to gain more from stronger evaluation than from another post hoc explainer. Priorities include cold-start and temporal splits, stability after retraining, provenance-aware external concordance, degree-matched null models, and a clear boundary between computational explanation and experimental confirmation.

Future methods should preserve relation semantics and expose bounded reasoning paths without sacrificing predictive usefulness. The decisive comparison is prospective: do explanation-guided experiments identify mechanisms more efficiently than prediction alone? Clinical use will require additional evidence, including externally demonstrated performance across populations and sites, explicit uncertainty, and an auditable chain connecting provenance, prediction, explanation, and intervention.

### Limitations of This Review

Three limitations shape how this review should be interpreted. First, it is a critical narrative review, not an exhaustive or systematic synthesis. The search was neither registered nor protocol-driven; the authors, rather than independent duplicate reviewers, performed screening; and selection was purposive. Relevant work may therefore have been missed, and selection and interpretation bias cannot be ruled out. Section 1.4 describes the search strategy, eligibility criteria, and these limitations. Second, the review generated no new empirical evidence. We did not analyze a dataset, train or rerun a model, perform quantitative benchmarking, or conduct a prospective experiment. Every classification therefore depends on what the cited publications report. Third, the five-domain framework, R1-R6 checklist, ordering rule in Section 9.2.4, and evidence categories in Section 9.2.5 remain conceptual. They have not been empirically evaluated, weighted, or calibrated; Section 9.5 explains what that process would require.

## 12. Conclusions

This review began with a straightforward problem: network pharmacology asks mechanistic questions, yet it increasingly relies on models whose internal reasoning is difficult to inspect. The literature considered here shows that explainability remains an unresolved methodological challenge, not a technical addition that can be taken for granted.

The technical landscape remains fragmented and immature. Feature-attribution methods carry assumptions that transfer poorly to heterogeneous, multi-hop biomedical graphs. Explainable-GNN approaches satisfy some of the graph-specific demands, though none satisfies all three. Causal and Bayesian methods address the multi-hop and uncertainty requirements more directly, although the reports identified here evaluate them on limited biomedical data. Knowledge-graph reasoning suits the field’s relational vocabulary particularly well because it is interpretable by design, an advantage that may be paid for in predictive flexibility.

Biological relevance poses a second difficulty. An explanation may be technically faithful and still say nothing useful about the mechanism. Degree shortcuts, hub over-attribution, and literature-popularity bias all arise from graph construction and message passing, and their presence has to be tested model by model. Left untested, an explainer can describe a shortcut accurately and lend it the appearance of biological insight.

The proposed evaluation framework offers a structured basis for comparing applications across faithfulness, stability, external biological concordance, bias sensitivity, and translational utility. Its status should not be overstated. The five domains, the R1–R6 checklist, the ordering rule for conflicting results, and the evidence categories are conceptual proposals by the present authors. They are unweighted, uncalibrated, and have not been empirically evaluated or calibrated. The assessment of TxGNN, DDI-GCN, and MINERVA in Section 9.4 is an exploratory, literature-based demonstration of how the criteria might be applied; no model was retrained or rerun, no new dataset was analyzed, and no quantitative benchmarking was carried out for this review. Section 9.5 sets out what empirical evaluation and calibration would require, and until that work is done the framework should be treated as a reporting aid and a basis for discussion rather than a calibrated instrument.

Its added value is therefore practical and scope-specific. It complements broad XAI taxonomies by providing a common audit trail for heterogeneous network-pharmacology claims, from method–task fit and model-level behavior to graph-bias controls, evidentiary classification, and the next falsifiable study.

Network pharmacology rests on the idea that disease and drug effects emerge from interacting systems rather than isolated nodes. Applying modern AI to that premise requires more than accurate prediction. Explanations must themselves be evaluated, alongside uncertainty, experimental evidence, and predictive performance. They must also be interpreted within their limits. An explanation indicates how a model arrived at an output; it can prioritize candidates and generate testable hypotheses, yet remain biologically wrong or clinically irrelevant even when it is faithful to the model. Computational and in vitro findings alone do not establish causality, efficacy, safety, or clinical relevance. Moving a model-derived rationale toward any of those claims requires independent experimental evidence.

## Figures and Tables

**Table 1:** Relationship between the 2026 broad XAI drug-discovery survey and the present network-pharmacology-specific contribution.

Comparison Point	Gangwal and Lavecchia (2026) [5]	Present Review	Practical Added Value in Network Pharmacology
**Primary purpose**	A multidimensional survey and taxonomy of XAI methods across drug-discovery applications.	A claim-level framework for auditing explanations produced from heterogeneous network-pharmacology models.	Moves from cataloging methods to specifying what should be reported for an individual graph-derived claim.
**Organizing unit**	Explanation methods considered across input modalities, transparency levels, and interpretability objectives.	A drug–target, drug–disease, protein-interaction, or multi-hop mechanistic claim represented on a biological graph.	Links method selection to the graph structure, prediction task, and evidentiary claim being made.
**Graph-specific semantics**	Graph explainers are considered within a broader drug-discovery taxonomy.	Typed nodes and relations, directionality, edge provenance, connectivity, and multi-hop path validity are explicit audit targets.	Makes preservation of biomedical relation meaning and path structure reportable rather than implicit.
**Evaluation criteria**	Discusses established concerns including fidelity, stability, sparsity, mechanistic plausibility, and task-dependent method choice.	Retains those concerns but organizes them with external biological concordance, bias sensitivity, and translational utility into five linked domains.	Prevents a strong result on one model-level metric from being treated as sufficient biological or translational support.
**Evidence boundary**	Relates interpretability to mechanistic insight across drug-discovery settings.	Uses Categories A-G to separate a model-derived explanation and database concordance from biochemical, cellular, animal, and human evidence.	Makes clear why agreement with STRING or Reactome is not experimental validation of a mechanism.
**Bias and provenance**	Addresses limitations and method-dependent considerations across XAI applications.	Foregrounds node degree, annotation density, literature popularity, source provenance, training-set overlap, and cold-start novelty as graph-specific checks.	Provides controls for common shortcuts in biomedical knowledge graphs, including degree-matched null comparisons.
**Reporting output**	A taxonomy that supports understanding and selection of XAI approaches.	Five evaluation domains plus an R1-R6 reporting checklist and evidence Categories A-G.	Ends with a stated evidentiary level, unresolved limitation, and the next study capable of supporting or falsifying the claim.
**Empirical status**	Literature-based review and synthesis.	Critical narrative review containing a conceptual, unweighted, and uncalibrated framework.	Defines a testable reporting and evaluation agenda without presenting the framework as already validated.

Note to Table 1: The comparison identifies differences in scope, organizing logic, and intended use. It does not claim that any of the individual XAI methods or evaluation concepts originated here. The framework itself is still conceptual, and it will need prospective evaluation and calibration.

**Table 2:** Task-based taxonomy of interpretability requirements in network pharmacology and the closest-fit XAI method families.

Task	What an Explanation Must Surface	Closest-Fit XAI Method Family
Drug–target prediction	Binding-relevant substructure or interaction motif	Feature attribution (SHAP, IG); GNNExplainer
Protein–protein interaction prediction	Interface residues/interaction-relevant subgraph	Subgraph explainers (SubgraphX, GraphMask)
Disease–gene association	Supporting path(s) through the knowledge graph	Path-based/interpretable KG reasoning
Drug repositioning	Mechanistic path linking existing drug to new indication	Path-based KG reasoning; counterfactual explanation
Polypharmacology/multi-target profiling	Which target subset drives the network-level effect	Attention mechanisms; SHAP on multi-task outputs
Biomarker/target prioritization	Node-level importance ranked against network centrality	Feature attribution; GraphLIME
Mechanism-of-action prediction	Full causal subnetwork, not just endpoint nodes	Causal explainability
Toxicity/ADMET prediction	Structural alerts or off-target network exposure	Feature attribution; counterfactual explanation

**Table 3:** Biomedical databases and knowledge graph platforms underlying network pharmacology pipelines, evaluated from an XAI-oriented perspective per the proposed bias-exposure framework.

Resource	Primary Content	Curation Model	XAI-Relevant Consideration
DrugBank [21]	Drug, target, and drug–gene interaction data	Manually curated	Well-studied approved drugs are heavily over-represented, a direct input to the literature-popularity bias of the failure-mode analysis
ChEMBL [22]	Bioactivity and compound data	Manually curated from the literature and patents	Assay-type heterogeneity can bias attribution methods toward well-assayed compound classes
BindingDB [23]	Measured binding affinities	Literature and depositor-curated	Overlaps substantially with ChEMBL; shared-source overlap can inflate apparent Category B cross-database concordance
DisGeNET [24]	Gene–disease associations	Text-mined and curated	Text-mining provenance makes literature-popularity bias especially likely to propagate into downstream GNNs
STRING [25]	Protein–protein association networks	Mixed: experimental, curated, and predicted	Predicted (non-experimental) edges are not flagged distinctly in most downstream uses, complicating faithfulness assessment
Reactome [26]	Curated pathway data	Expert-curated	High curation quality but incomplete coverage biases path-based explanations toward well-characterized pathways
STITCH [27]	Chemical–protein interaction networks	Mixed: experimental and predicted	Predicted-interaction inflation risk analogous to STRING
Open Targets [28]	Target–disease association scores	Aggregated from multiple evidence types	Evidence-type weighting is itself a modeling choice that can silently bias downstream target prioritization
Hetionet [19]	Integrated heterogeneous biomedical graph (24 edge types)	Aggregated from 29 public resources	Aggregation inherits every contributing resource’s individual biases simultaneously
PrimeKG [13]	Precision-medicine-oriented knowledge graph	Aggregated, disease-centric	Designed with repositioning tasks in mind; benchmark splits should be checked for the cold-start rigor of the failure-mode analysis
CTD (Comparative Toxicogenomics Database) [29]	Chemical–gene–disease interactions	Manually curated from the literature	Toxicology-oriented curation focus may under-represent efficacy-relevant mechanisms

**Table 5:** How XAI method families considered above address (or fail to address) the three concrete failure modes identified above.

Failure Mode	Feature Attribution	Attention	Explainable GNNs	Causal-GNN	Path-Based KG Reasoning
Degree-bias shortcut learning	Cannot detect	Cannot detect	Cannot detect	Partially addresses	Makes visible for inspection
Hub over-attribution	Inherited from model	Directly exhibits	Directly exhibits	Reduced if well-specified	Reduced path specificity explicit
Literature-popularity bias	Cannot detect	Cannot detect	Cannot detect	Cannot detect	Surfaces which edges used

**Table 6:** Benchmark datasets relevant to evaluating explanation stability, entity novelty, and sensitivity to graph structure.

Dataset	Task	Relevance to the Evaluation Framework Dimensions
Therapeutics Data Commons (TDC) [65]	22 learning tasks across drug discovery/development	Provides standardized cold-start, scaffold, and temporal splits directly usable for the bias-sensitivity domain
DAVIS [66]	Kinase inhibitor binding affinity (72 inhibitors × 442 kinases)	Small, well-characterized; useful for stability checks across resampling
KIBA [67]	Kinase inhibitor bioactivity (2111 inhibitors × 229 kinases)	Larger-scale kinase DTI benchmark; commonly paired with DAVIS for cross-dataset stability checks
BioSNAP [68]	Biological network datasets (drug–drug, drug–target, PPI)	Network-native benchmark suitable for testing degree-bias exposure directly on graph structure
PrimeKG-derived repurposing benchmarks	Zero-shot drug repurposing	Explicitly designed with disease-centric zero-shot splits; directly tests cold-start robustness

**Table 7:** Metrics for evaluating explanation faithfulness, compactness, stability, and external biological concordance.

Metric or Criterion	Operational Definition	Required Parameters	Interpretation and Principal Limitation	Source
Deletion-based fidelity	Change in model output when the explanatory subgraph is removed	Perturbation unit, output scale, replacement baseline, aggregation rule, and full-mask baseline sanity check	A large change supports necessity. Sign and scale conventions differ and the result is baseline-sensitive.	[6,7]
Retention-based fidelity	Model output when only the explanatory subgraph is retained	Perturbation unit, handling of discarded elements and aggregation rule	Similarity to the original output supports sufficiency. Large retained subgraphs can inflate performance.	[6,7]
Characterization score	Combination of deletion- and retention-based fidelity	Definitions and weighting of both fidelity components	Distinguishes sufficient from necessary explanations, but inherits both components’ sensitivities.	[6]
Sparsity	Fraction of nodes or edges retained relative to a stated denominator	Node, edge or computation-graph denominator	Lower values indicate compactness, but sparsity must be interpreted jointly with fidelity.	[6,7]
Ground-truth motif agreement	Overlap with an independently annotated structural motif	Annotated motif and overlap statistic	Supports recovery of known chemistry, but suitable ground truth is rare for heterogeneous biomedical graphs.	[8]
Stability	Agreement across retraining, resampling or small graph perturbations	Number of runs, perturbation magnitude and rank- or set-agreement statistic	Higher agreement indicates lower sensitivity to optimization or sampling variation. No universal threshold is established.	Author-proposed extension; Section 9.2
External biological concordance	Agreement with independent resources after removal of training-source overlap	Identifier mapping, exclusion procedure, external resources and degree-matched null	Indicates agreement with curated knowledge, not experimental confirmation; results are coverage- and popularity-sensitive.	Author-proposed extension; Section 9.2
Subgraph connectivity	Whether the retained nodes and edges form a connected subgraph	Explanatory edge set	Connectivity supports structural coherence but is descriptive and does not establish mechanism.	Author-proposed extension; Section 9.2.3
Path validity	Whether edge directions and relation semantics compose into a traversable path	Directed, typed edge set	Supports interpretation as a candidate route; depends on consistent source-graph annotation.	Author-proposed extension; Section 9.2.3
Relation-type preservation	Whether retained edges preserve their reported biological relation types	Typed edge set and provenance	Enables biological interpretation; limited by the quality of source annotations.	Author-proposed extension; Section 9.2.3
Degree-matched null comparison	Comparison with random subgraphs matched for size and degree profile	Null-sampling procedure, matching rules and number of samples	A score not exceeding the null is compatible with a structural artifact; null construction is itself a modeling choice.	Author-proposed extension; Section 9.2.3

Note to Table 7: The metrics listed are drawn from published sources, and the entries describe what each metric measures rather than reporting values computed by the present authors; no metric in this table was calculated for this review. Operational definitions, required parameters, and interpretive limitations for each metric are given in Section 9.2 and Section 9.2.3, and readers implementing them should consult the cited primary sources [6,7,8] for the exact formulation, since sign conventions and perturbation rules differ between implementations. The proposed domains and checklist have not been prospectively evaluated, weighted, or calibrated against experimental success. External-knowledge concordance may reward well-studied mechanisms, explanation metrics depend on perturbation and sign conventions, and no single benchmark represents the heterogeneity of pharmaceutical knowledge graphs. The framework should therefore be treated as a basis for comparative testing and community refinement rather than as a certification instrument.

**Table 8:** Exploratory, literature-based application of the proposed reporting and evaluation criteria to TxGNN, DDI-GCN, and MINERVA. This is a demonstration of how the criteria could be applied and is not an empirical evaluation or calibration of the framework.

Criterion	TxGNN [10]	DDI-GCN [11]	MINERVA [12]
NP2-P1: Structural interpretability	Partially addressed: explainer returns sparse subgraphs and multi-hop paths	Partially addressed: feature- and graph-based rationale reported for DDI predictions	Reported: returns an explicit reasoning path by construction
NP2-P2: Multimodal grounding	Partially addressed: integrated medical knowledge graph; no independent non-graph modality used for grounding	Partially addressed: chemical structure and graph information combined; no independent foundation-model check reported	Not applicable to original scope: evaluated on general knowledge graphs, not biomedical modalities
NP2-P3: Evidentiary discipline	Partially addressed: human evaluation and comparison with medical evidence; no interventional evidence reported	Partially addressed: mechanistic interpretation presented as observational	Not applicable to original scope: paths are predictive; no causal claim made
NP2-P4: Governed trustworthiness	Partially addressed: zero-shot disease evaluation reported; no degree, provenance, or subgroup audit reported	Partially addressed: predictive benchmarking reported; no bias-stratified analysis reported	Partially addressed: benchmark evaluation reported; biomedical bias audit outside original scope
NP2-P5: Translational accountability	Partially addressed: clinician-facing interface and candidate rationales; no prospective clinical evaluation reported	Partially addressed: adverse-DDI interpretation offered; no prospective translational evaluation reported	Not applicable to original scope: general knowledge-graph reasoning method
R1: Method and task justification	Reported	Reported	Reported
R2: Standardized faithfulness metric	Not reported: no harmonized biomedical faithfulness protocol applied	Not reported: no harmonized GNN-XAI faithfulness protocol applied	Not reported in the primary publication
R3: Stability across seeds/perturbations	Not reported for explanations	Not reported for explanations	Not reported: no stability analysis for reasoning paths
R4: External biological concordance	Partially addressed: expert review and medical-evidence case studies	Partially addressed: qualitative interpretation of DDI mechanisms	Not applicable to original scope: no biomedical evaluation performed
R5: Bias-sensitivity analysis	Partially addressed: zero-shot disease splits reported; degree and data-source bias not tested	Not reported in the primary publication	Not reported in the primary publication
R6: Limits, evidence category, and next evidentiary step	Partially addressed	Partially addressed	Partially addressed: limits stated for general reasoning; biomedical evidentiary level not specified

With these qualifications in mind, the three publications show a common pattern. Each makes some part of its reasoning inspectable, but none reports the complete combination of standardized faithfulness, stability, external concordance, bias sensitivity, and translational evidence. We take this to suggest that such reporting has not yet become routine, rather than that the individual studies are deficient. A numerical ranking would be misleading because the systems address different tasks, use different data structures, and answer different evidentiary questions. The R1–R6 checklist is intended to make these differences more transparent. Note to Table 8: The classifications in this table are based exclusively on the methods, results, and evidence reported in the cited primary publications [10,11,12]. The authors of the present review did not rerun the models, access their underlying data, or generate new benchmarking results. “Reported” indicates that the primary publication presents information meeting the criterion. “Partially addressed” indicates that the publication addresses the criterion but does not cover all of its elements as proposed here. “Not evaluated in the original study” and “Not reported” indicate an absence of information in the publication; neither should be read as evidence that a procedure was not performed, nor as criticism of the study’s original objectives. “Not applicable to original scope” indicates that the criterion falls outside the problem the study set out to address. All three publications predate the criteria proposed in this review. The table is an exploratory application of a conceptual framework and does not constitute empirical evaluation or calibration of it.

## Data Availability

No new data were created or analyzed in this study. Data sharing is not applicable to this article.

## Appendix A

Table A1 maps the representative primary studies used in the methodological comparison. Because this is a critical narrative review, the table documents the purposively selected evidence base rather than offering a complete inventory of all relevant studies.

**Table A1:** Representative primary studies anchoring the comparison of explanation methods and biomedical graph applications.

Study or Method	Graph or Data Setting	Explanation Output	Role in the Present Synthesis
GNNExplainer [34]	General graph neural networks	Feature and edge masks forming a subgraph	Foundational post hoc subgraph explanation
PGExplainer [35]	General graph neural networks	Parameterized edge or subgraph scores	Shared explainer and scalability comparison
SubgraphX [36]	General and molecular graphs	Connected explanatory subgraph	Search-based explanation and computational scaling
GraphMask [37]	Message-passing neural networks	Layer-specific edge masks	Differentiable masking and hop-level interpretation
GraphLIME [38]	Node-level graph prediction	Local node-feature relevance	Local surrogate explanation on graphs
CF-GNNExplainer [39]	Graph neural networks	Minimal counterfactual graph edits	Counterfactual testing of prediction sensitivity
PGM-Explainer [40]	Graph neural networks	Local probabilistic dependency graph	Probabilistic explanation and residual uncertainty
MINERVA [12]	Multi-relational knowledge graphs	Ordered reasoning path	Path-based, relation-aware reasoning
DeepPath [41]	Knowledge graphs	Reinforcement-learned relation path	Multi-hop path reasoning comparison
DDI-GCN [11]	Drug–drug interaction graph	Interpretable graph-convolutional rationale	Biomedical application of explainable graph prediction
TxGNN [10]	Heterogeneous biomedical knowledge graph	Explainer-supported drug-repurposing rationale	Network-pharmacology application and reporting demonstration
Sudhahar et al. [53]	Drug-discovery knowledge graph	Automated evidence linked to experiments	Connection between graph hypotheses and direct experimental support
GraphFramEx and related evaluations [6,7,8]	Synthetic, general, and molecular graph benchmarks	Fidelity, sparsity and motif-level measures	Foundation for the operational evaluation domains
